# *Lactobacillus acidophilus* ameliorates cholestatic liver injury through inhibiting bile acid synthesis and promoting bile acid excretion

**DOI:** 10.1080/19490976.2024.2390176

**Published:** 2024-08-29

**Authors:** Lingyi Wu, Jianchun Zhou, An Zhou, Yuanyuan Lei, Li Tang, Shiping Hu, Sumin Wang, Xu Xiao, Qiao Chen, Dianji Tu, Cheng Lu, Yi Lai, Yiding Li, Xiao Zhang, Bo Tang, Shiming Yang

**Affiliations:** a Department of Gastroenterology, Xinqiao Hospital, Third Military Medical University, Chongqing, China; b Shigatse Branch, Xinqiao Hospital, Third Military Medical University, Tibet, China

**Keywords:** Cholestatic liver injury, gut microbiota, Lactobacillus

## Abstract

Gut microbiota dysbiosis is involved in cholestatic liver diseases. However, the mechanisms remain to be elucidated. The purpose of this study was to examine the effects and mechanisms of *Lactobacillus acidophilus* (*L.* *acidophilus*) on cholestatic liver injury in both animals and humans. Bile duct ligation (BDL) was performed to mimic cholestatic liver injury in mice and serum liver function was tested. Gut microbiota were analyzed by 16S rRNA sequencing. Fecal bacteria transplantation (FMT) was used to evaluate the role of gut microbiota in cholestasis. Bile acids (BAs) profiles were analyzed by targeted metabolomics. Effects of *L.* *acidophilus* in cholestatic patients were evaluated by a randomized controlled clinical trial (NO: ChiCTR2200063330). BDL induced different severity of liver injury, which was associated with gut microbiota. 16S rRNA sequencing of feces confirmed the gut flora differences between groups, of which *L.* *acidophilus* was the most distinguished genus. Administration of *L.* *acidophilus* after BDL significantly attenuated hepatic injury in mice, decreased liver total BAs and increased fecal total BAs. Furthermore, after *L.* *acidophilus* treatment, inhibition of hepatic Cholesterol 7α-hydroxylase (CYP7α1), restored ileum Fibroblast growth factor 15 (FGF15) and Small heterodimer partner (SHP) accounted for BAs synthesis decrease, whereas enhanced BAs excretion was attributed to the increase of unconjugated BAs by enriched bile salt hydrolase (BSH) enzymes in feces. Similarly, in cholestasis patients, supplementation of *L.* *acidophilus* promoted the recovery of liver function and negatively correlated with liver function indicators, possibly in relationship with the changes in BAs profiles and gut microbiota composition. *L.* *acidophilus* treatment ameliorates cholestatic liver injury through inhibited hepatic BAs synthesis and enhances fecal BAs excretion.

## Introduction

Disorders in BAs homeostasis characterize cholestasis. Disrupted transport and metabolism of BAs are responsible for nonalcoholic fatty liver disease, liver fibrosis and cirrhosis.^1–3^ During cholestasis, overload BAs in the liver play a vital role in pathogenesis. Due to poor prognosis, cholestatic liver diseases become a major burden on the overall quality of life. Therefore, there is an urgent need for efficient treatment to address cholestatic liver injury effectively.

Gut microbiota, as part of the host immune system, plays an essential role in regulating host health. Given the liver and intestine are identical in embryonic origin and maintain a natural physiological functional link, the contribution of the gut-liver axis is non-negligible.^4,5^ Multiple substances are metabolized, including BAs,^6,7^ short-chain fatty acids (SCFAs),^8^ vitamins and neurotransmitters.^9^ Increasing evidence indicates that gut microbiota is closely related to cholestatic liver injury progression and therapeutic effect.^10–13^ Dysfunction of gut microbiota is involved in intestinal inflammation and barrier impairment, thereby aggravating liver injury.^11^ Supplementation with *Lactobacillus rhamnosus GG* (LGG) can activate the expression of nuclear factor erythroid-derived 2-like-2 (Nrf2) in the liver and prevent liver damage and fibrosis.^14^ In addition, crosstalk between gut microbiota and BAs metabolism is highly meaningful in cholestatic liver injury.^6^ However, the specific gut microbe and the underlying mechanism by which the gut microbiota impact cholestatic liver injury are still unknown.

Here, we aim to explore whether the gut microbiota has an important role in BDL-induced liver injury and clarify the potential underlying mechanism. Our study showed that *Lactobacillus* was significantly reduced in severe cholestatic liver injury mice. Furthermore, we discovered the therapeutic effect of *L. acidophilus* on cholestatic liver injury and elucidated that *L. acidophilus* inhibited hepatic BAs synthesis by activating intestinal Farnesoid X receptor (FXR) signaling, and enhanced the BAs excretion. Our data unveils the importance of specific gut microbiota in the progression and treatment of cholestatic liver injury as well as providing a potential target for the clinical management of cholestatic liver injury.

## Materials and methods

### Animal study

All animal procedures and testing were approved by the Laboratory Animal Welfare and Ethics Committee of Third Military Medical University (Chongqing, China) on Investigations Involving Animal Subjects (No. AMUWEC20224566). Male C57BL/6J mice (6–8 weeks of age) were obtained from the Animal Center of Xinqiao Hospital. Prof. J.C. from the Department of Gastroenterology, Southwest Hospital (Third Military Medical University) provided the Mdr2^−/−^ mice. They were maintained at 22°C with a 12-hour:12-hour light:dark cycle and had free access to a normal chow diet and sterile water. The experiment was conducted after 7 days of adaptive feeding. Clean standard feed was provided throughout. Cholestatic liver injury was induced by BDL as previously described.^15,16^ Briefly, mice were anesthetized with isoflurane avertin inhalation. After laparotomy, the bile duct was double-ligated with nonresorbable surgical sutures. The sham group underwent the same surgical procedure except for ligation. Once it was confirmed that there was no bleeding or injury of the viscera in the abdominal cavity, the abdomen was closed. For postoperative recovery, the mice were placed in a clean and warm environment.

### Serum biochemical value analysis

Serum was collected by centrifugation from whole blood samples at 4000 × g for 30 min at room temperature. Serum alanine aminotransferase (ALT), aspartate aminotransferase (AST), total bile acid (TBA), alkaline phosphatase (ALP), total bilirubin (TBIL) and γ-glutamyl transpeptidase (GGT) levels were measured by using standard laboratory assays.

### FMT

FMT was performed according to the modified method described.^17,18^ Briefly, 6–8 weeks of male C57BL/6 mice received antibiotics (vancomycin,100 mg/kg; neomycin sulfate, 200 mg/kg; metronidazole, 200 mg/kg; and ampicillin, 200 mg/kg) (Cat# A600633, Cat# A610366, Cat# A610028 and Cat# A600983, Sangon, Shanghai, China) intragastrically once a day for 5 days to deplete the gut microbiota. Feces from donor mice (severe and mild mice) were collected and resuspended in PBS at 0.125 g/mL, and 0.15 mL suspension was administered to mice by oral gavage once a day.

### Quantitative reverse transcription-polymerase chain reaction (qRT-PCR)

A TRizonRNA Extract kit (Cat# P2002, Engreen, Beijing, China) was used to extract total RNA. The PrimeScript RT reagent kit (Cat# RR047A, Takara, Kusatsu, Japan) was used to synthesize cDNA. qRT-PCR was conducted with a TB Green Premix Ex Taq II kit (Cat# RR820A, Takara). Primers for the experiment are listed in Supplementary Table 1. 18S or GAPDH were used as controls. The relative gene expression was determined by the 2-ΔΔCT method.

### Microbial strain

*L. acidophilus* was purchased from American Type Culture Collection (ATCC, Cat# 4356, USA) and cultured in MRS medium (Cat# 8540, Solarbio, China) under 37°C anaerobic incubator for 48 h (GeneScience, E500, USA). The number of living cells was determined by counting the colony-forming units (CFUs). Live *L. acidophilus* was given to mice by oral gavage at a dose of 10^8^ CFU/200 μl/day for 9 days. One fresh aliquot was thawed for every new experiment to avoid variability in the viability of cultures. The colonization of *L. acidophilus* was confirmed by qRT-PCR^19^(Supplementary Table 2). *Lactobacilli* species probes are labeled with the 5 reporter dye 6-carboxy-fluorescein (FAM) and the 3 quencher NFQ-MGB (Sangon Biotech, China) by TaqMan minor groove binding probes.^20,21^ qRT – PCR was performed using the StepOnePlus system (Applied Biosystems) based on TaqMan detection. Each sample contained 5 µl of DNA template, 10 µl Taq polymerase, 1 µl of each universal primer (10 µmol), 1 µl of TaqMan® labeled specific primer (10 µmol, each 6-FAMTM/Dabcyl-labeled) and 2 µl of PCR grade water. A single initial denaturation step of 10 min at 95°C was followed by 40 cycles of 95°C for 1 min (denaturation), 53°C for 30 s (annealing) and 73°C for 30 s (elongation). The fluorescence signal was measured at the end of each 73°C elongation step.

### Fecal sample collection and fecal genomic DNA extraction

Fecal samples (100 mg) from humans or mice were separately collected. Fecal genomic DNA with the TIANamp Stool DNA kit (Cat# DP328–02, Tiangen, China). The DNA purity and concentration were measured with a NanoDrop 2000C spectrophotometer (Thermo Scientific, USA).

### 16S rRNA gene sequencing

The primers specific to the V3-V4 region of the 16S rRNA gene were 341F (CCTAYGGGRBGCASCAG) and 806 R (GGACTACNNGGGTATCTAAT). Products were purified quickly and efficiently for library construction using the GeneJET Gel Extraction Kit (Cat# K069, Thermo Scientific, USA), and all libraries were sequenced using the Illumina NovaSeq 6000 platform from Novogene. The next step of analysis was performed after the return of Raw data. Sequencing data were processed in conda environment using QIIME2 (version number: 2020.2) platform. Briefly, the V3~V4 primers of the double-ended fastq format sequence file were cut in Python 3.6.9 using the cutadapt 3.1 command. In this way, the cut fastq files were imported into QIIME2 using the “qime tools import” command, and the reads were denoised into amplicon sequence variants (ASVs), and each ASV was generated. A representative sequence and feature table for each ASV are generated simultaneously. Qzv files were generated and samples with depth less than 8000 were excluded (non-chimeric sequences). Refine all remaining samples to the same depth and a granulated feature table is created. Calculate and visualize diversity from the refined feature table. Accomplish ANOSIM for the significance of the differences between sample groups. LEfSe analysis the dominant bacteria in the bacterial community between groups (LDA score (log10) = 2 as cutoff value).

### Targeted BA metabolism

For bile acid analysis, blood samples were obtained after an overnight fast of 10 h, and serum was obtained by centrifugation (4000 g, 10 min). 50 μL of serum was mixed with 150 μL of methanol. The supernatant was vortexed for 2 min, centrifuged at 20,000 × g for 10 min at 4°C, and dried under vacuum to 160 μL. The supernatant was redissolved with acetonitrile and water to a volume of 40 μL. Supernatant was used for UPLC-MS/MS (Waters Corp., USA) analysis. 10 mg of liver/fecal sample was added with 200 μL of methanol/water (1:1). The samples were homogenized and centrifuged at 13,000 × g for 15 min, and the supernatant was pipetted into a tube and the sample residue was extracted with methanol/acetonitrile (2:8). The extract was vortex centrifuged at 13,000 × g for 15 min and the supernatant was taken for further analysis. Bile acids were analyzed by UPLC-MS/MS (Waters Corp., USA). The elution solvents were water + 0.01% formic acid (A) and acetonitrile/methanol (19:1) + 0.01% formic acid (B), and the elution gradients at a flow rate of 450 μL/min were: 0–2 min (20% B), 2–3 min (20–25% B), 3–6 min (25% B), 6–8 min (25–35% B), 8–11.5 min (35% B), 11.5–18 min (35–99% B), 18–19 min (99% B), 19–20 min (99–20% B), with peaks annotated and quantified by TargetLynx Application Manager. Multi quant 2.1 software was used for bile acid data collection.

### A single-center randomized controlled clinical trial

This study was approved by the Medical Ethics Committee of Xinqiao Hospital (Chongqing, China) (Approval number. 2022-019-01) and has also been registered with the Chinese Clinical Trials Registry (ChiCTR) of the World Health Organization Registry Network (Registration number: ChiCTR2200063330). The study conforms to the CONSORT statement requirements. This clinical trial was conducted following the principles of the Declaration of Helsinki. All volunteers provided written informed consent before participation in this study. The manuscript was written under ICMJE guidelines.

#### Subjects

Twenty patients with cholestatic liver disease were recruited from the Department of Gastroenterology, Xinqiao Hospital (Chongqing, China). Inclusion criteria^1^: Over 18 years and a clear diagnosis of cholestatic liver disease (ALP ≥1.5 times the upper limit of normal value and GGT ≥ 3 times the upper limit of normal value) and^2^ willingness to participate in this study and to sign an informed consent form. Exclusion criteria^1^: previous use of microecological agents within 14 days before enrollment^2^; the presence of chronic infections (e.g., tuberculosis, HIV infection, parasitic infections, etc.)^3^; the presence of neurological or psychiatric disorders that could prevent effective provision of informed consent or that may interfere with the subject’s compliance with study procedures (e.g., major depression within the last 2 years, a history of suicidal behavior within the last 3 months, etc.)^4^; a history of other psychiatric disorders, including schizophrenia or bipolar disorder^5^; Use of multiple medications for the treatment of illness in 1 month, such as antibacterial drugs, antacids, bismuth, tannins, medicinal charcoal, tinctures, etc.;^6^ pregnancy or lactation; and^7^ failure to sign the informed consent form and/or to not complete the study procedure in its entirety. All volunteers were recruited by the investigators. Participants did not receive cash compensation. However, the study administrators were responsible for registration fees and therapy examinations. Baseline characteristics are shown in Supplementary Table 3.

#### Study procedure and analysis

For this clinical trial, 20 subjects were randomly divided into two groups, treatment group received ursodeoxycholic acid (UDCA) (250 mg bid, Losan Pharma GmbH, Ursofalk, German) + *L*. acidophilus tablets (1 g of a fixed dose tablets before each meal and three times a day, Tonghua Golden-horse Group, China), the control group only received UDCA. 14 days afterward, human blood (collected after 10 h of fasting) and stool samples (stored at −80°C) were collected. Clinical indicators such as AST, ALT, ALP, GGT, and TBIL were measured at Xinqiao Hospital (Chongqing, China). Fecal 16S rRNA sequencing was performed by Novogene. Targeted BAs sequencing was performed by Panomix.

#### Outcomes

The primary aim was to describe changes in liver function, BAs profile, and diversity of gut microbiota of treatment with *L*. acidophilus tablets.

### Statistical analysis

The statistical analysis method to determine the significance level was selected based on whether the data were normally distributed. GraphPad Prism 8.0 software (GraphPad Software Inc., San Diego, USA) was used for the T-test (unpaired, two-tailed, or Mann-Whitney test and Wilcoxon matched-pairs signed rank test) to determine the significance level of comparisons between two groups. The results were presented as the mean ± standard error of the mean (SEM). The statistical significance was *p < 0.05; * *p < 0.01; ***p < 0.001; * * * *p < 0.0001, and ns indicates no significance.

## Results

### Characterization of mice with BDL-induced injury

**Figure 1.**
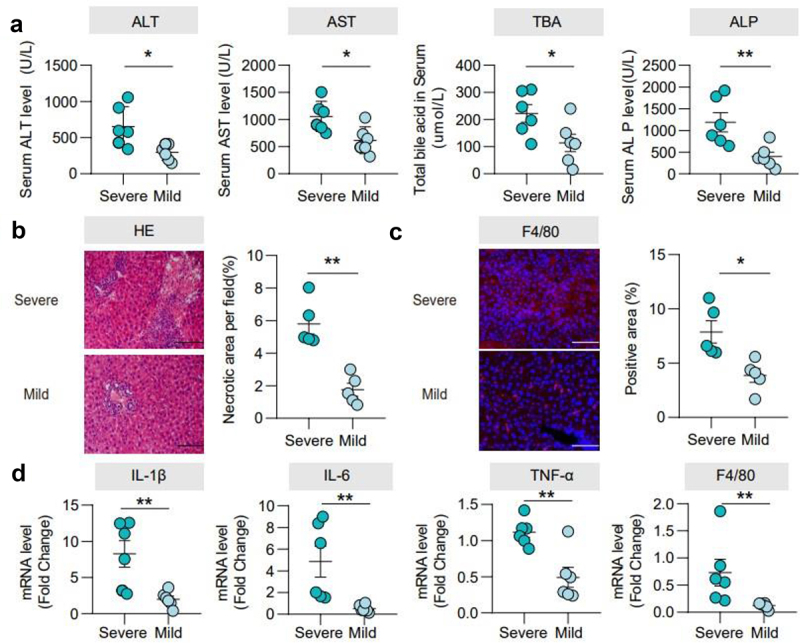
Characterization of mice with BDL-induced injury. (a). Serum ALT, AST, TBA, and ALP levels. (b). Representative images of the liver stained with hematoxylin and eosin (100 μm) and the quantification of the liver cell necrotic area. (c). Representative images of immunofluorescence analyses of F4/80 (200μm) and the quantification of the positive area. (d). Hepatic mRNA expression of inflammation-related genes. *n* = 6 individuals/group. Data are expressed as the mean ± SEM. Two-tailed Student’s t-test or Mann–Whitney test. Columns with different letters differ significantly (*P* < 0.05). *P < 0.05, **P < 0.01. Scale bar: 100 μm or 200 μm.

By observing the postoperative performance of BDL mice, we found that the median survival was 5 days (Supplementary Figure 1a). We differentiated the BDL mice into severe or mild group based on the survival time. The severe group exhibited higher levels of ALT, AST, TBA, ALP, and TBIL in serum than the mild group (Figure 1(a), Supplementary Figure 1b). Hematoxylin and eosin (H&E) staining indicated more hepatocyte necrosis (Figure 1(b)). Additionally, compared to the mild mice, the severe group expressed higher inflammatory levels of F4/80 protein (Figure 1(c)), and the mRNA level of F4/80, tumor necrosis factor α (TNF-α), interleukin-6 (IL-6), and interleukin-1β (IL-1β) is increased more significantly (Figure 1(d)). Severe mice also had a higher liver/body weight (%) (Supplementary Figure 1C), indicating that mice have different tolerances to liver injury after BDL.

### The severity of cholestatic liver injury depends on the transmission of gut microbiota

**Figure 2.**
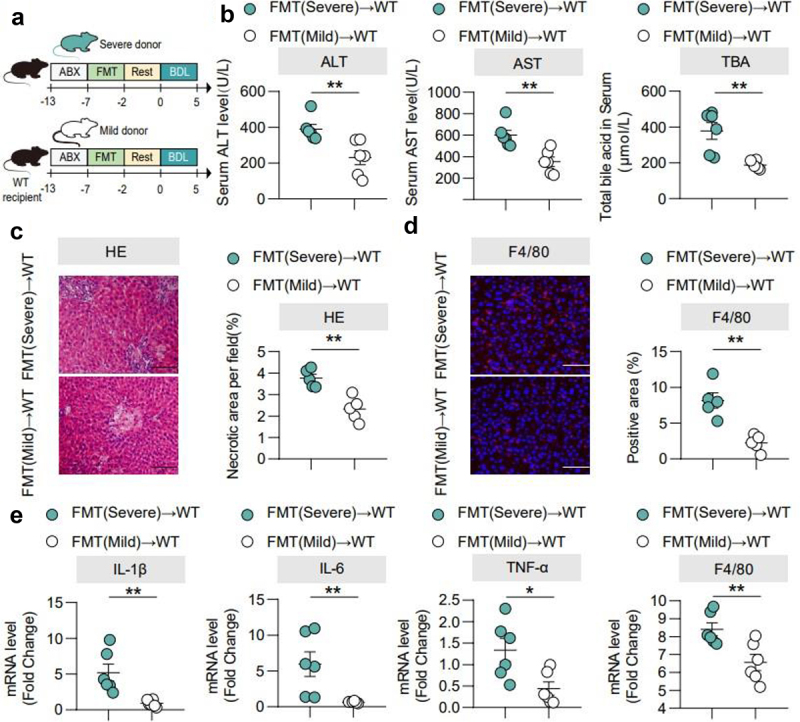
The severity of cholestatic liver injury depends on the transmission of gut microbiota. (a). FMT experimental design: mice received vancomycin (100 mg/kg), neomycin sulfate (200 mg/kg), metronidazole (200 mg/kg), and ampicillin (200 mg/kg) intragastrically once daily for 5 days to deplete the gut microbiota, in which feces sample derived from BDL-severe and BDL-mild and sacrificed at 5 days after BDL. (b). Serum ALT, AST, and TBA levels. (c). Representative images of liver stained with hematoxylin and eosin (100 μm) and quantification of liver cell necrosis area. (d). Representative images of immunofluorescence analyses of F4/80 (200 μm) and the quantification of the positive area. (e). Hepatic mRNA expression of inflammation-related genes. *n* = 6 individuals/group. Data are expressed as the mean ± SEM. Two-tailed Student’s t-test or Mann–Whitney test. Columns with different letters differ significantly (*P* < 0.05). *P < 0.05, **P < 0.01. Scale bar: 100 μm or 200 μm. Abbreviations: WT, wild type.

To verify whether gut microbiota is involved in cholestatic liver injury progression, we pre-treated wild-type (WT) mice with an antibiotic cocktail (ABX) for 5 days, feces from previously described BDL-mild or BDL-severe mice were used for FMT before BDL surgery (Figure 2(a)). Interestingly, FMT (Mild) group showed significantly lower liver/body weight (%) (Supplementary Figure 2(b)), serum AST, ALT, TBA, TBIL (Figure 2(b), Supplementary Figure 2a) and less hepatocyte necrosis (Figure 2(c)) than FMT (Severe) group. Hepatic mRNA levels of TNF-α, IL-1β, IL-6 and F4/80 were higher in the FMT (Severe) transplantation group (Figure 2(d,e)). These data indicated that the severity of cholestatic liver injury was dependent upon gut microbiota.

### Different BDL groups showed differences in gut microbiota

**Figure 3.**
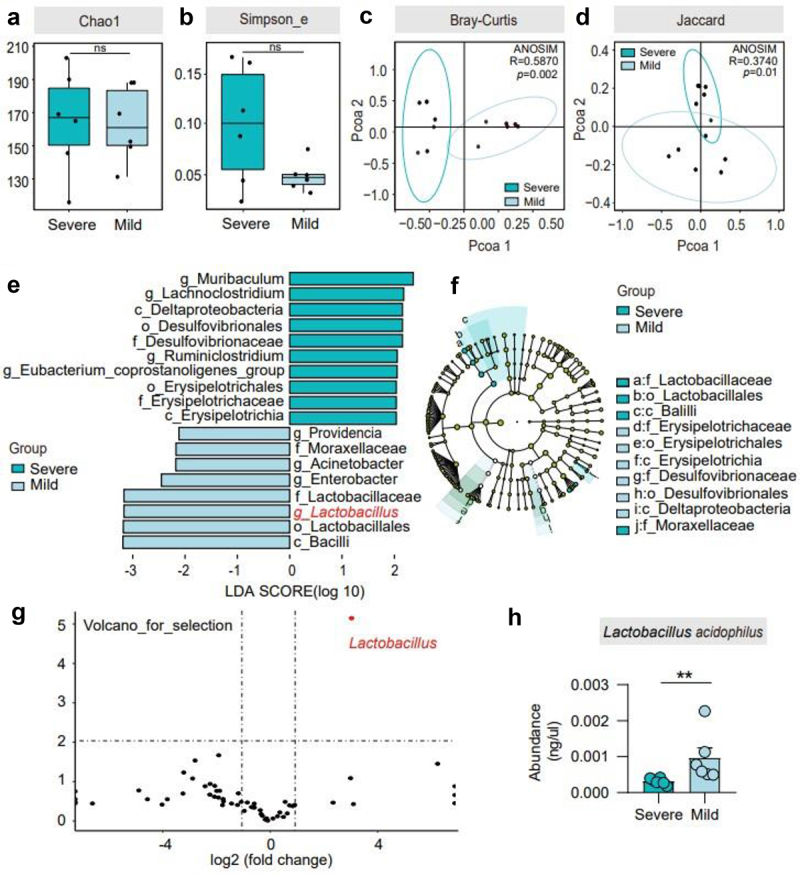
Different BDL groups showed differences in gut microbiota. (a)-(b). Alpha diversity in the two groups, according to the Chao1 and Simpson_e diversity indices. (c)-(d). Bray–Curtis and Jaccard distances from the two groups were determined by unweighted UniFrac PCoA (principal coordinates analysis) of the gut microbiota. (e)-(f). Cladogram using the LDA model results for the bacterial hierarchy. Differences were represented by the color of the most abundant class. The diameter of each circle is proportional to the abundance of the taxon. Each ring represents the next lower taxonomic level. LEfSe analysis indicated genera strikingly different among the gut microbiota. (g). Volcano plot displaying the relative abundance distribution of microbial OTUs. X-axis, log2 relative abundance; Y-axis, microbial OTU%. Each symbol represents one mouse or bacterial taxa. (h). The abundance of *L. acidophilus* in the severe and mild groups is shown. *n* = 6 individuals/group. For (c and d), differences in data were assessed by the ANOSIM test. Exact *p* levels are provided for all. Two-tailed Student’s t-test. Columns with different letters differ significantly (*P* < 0.05). * *P* < 0.05, ** *P* < 0.01. Abbreviations: OTUs, operational taxonomy units; ANOSIM, analysis of similarities; LDA, linear discriminant analysis; LEfSe, linear discriminant analysis effect size; PCoA, principal coordinate analysis.

To better understand the gut microbiota composition, 16S rRNA sequencing of feces from severe and mild mice was performed. There was no difference in α-diversity between the two groups (*p* > 0.05) (Figure 3(a,b)). The β diversity showed a significant difference in the composition of gut flora (*p* ≤ 0.01) (Figure 3(c,d)). At the phylum level, both groups were enriched mostly with *Bacteroidetes*, *Firmicutes* and *Proteobacteria*. *Bacteroidetes* accounted for 36.6% and 24.9% in the severe and mild groups, respectively; *Firmicutes* for 33.7% and 47.3%, respectively; and *Proteobacteria* accounted for 22.8% and 22.9%, respectively. At the genus level, the severe group was enriched with *Alloprevotella*, *Enterococcus*, *Escherichia_Shigella*, *Helicobacter* and *Lachnospiraceae_NK4A136_group*, while the mild group was enriched with *Enterobacter*, *Lactobacillus* and *Ruminococcaceae_UCG_014* (Supplementary Figure 3a,b).

To further confirm the presence of a key genus potentially affecting BDL-induced cholestatic liver injury, we performed a high-dimensional taxonomic comparison using linear discriminant analysis (LDA) with effect size (LEfSe), the results suggested that *Lactobacillus* was signed between two groups (Figure 3(e,f)). A volcano plot also flagged *Lactobacillus* genus as the key factor (Figure 3(g)). Consistently, the heatmap showed that the abundance of *Lactobacillus* is higher in the mild group (Supplementary Figure 3c). In addition, we performed PCR amplification on common *Lactobacillus* species (Supplementary Figure 3d) and found that *L. acidophilus* had higher levels in the mild group (Figure 3(h)).

### Administration of L. acidophilus ameliorated cholestatic liver injury in cholestasis mice

**Figure 4.**
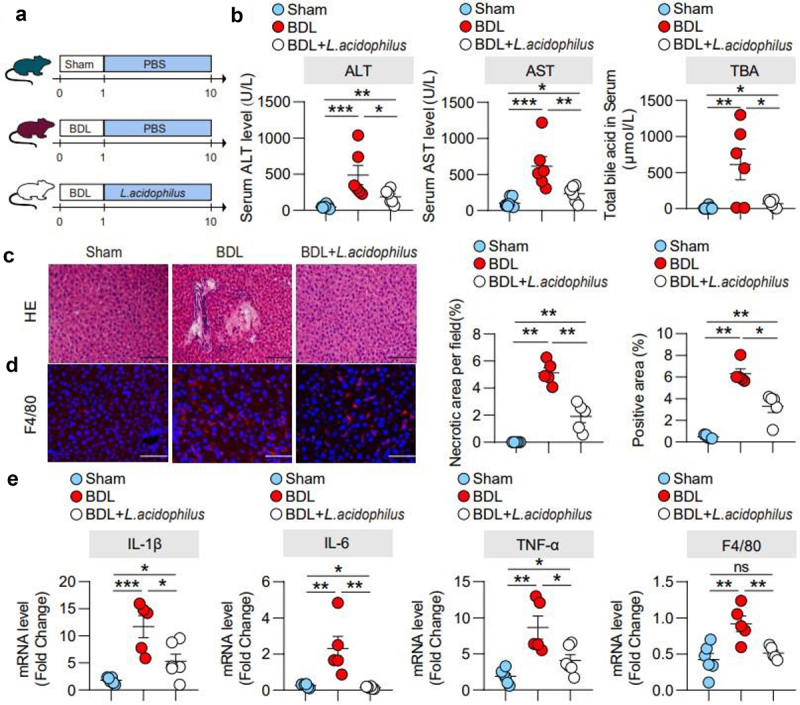
Administration of *L. acidophilus* ameliorated cholestatic liver injury in cholestasis mice. (a). BDL was completed after one week of adaptive feeding, and *L. acidophilus* was administered for 9 days before sacrifice. (b). Serum ALT, AST and TBA levels. (c). Representative images of liver stained with hematoxylin and eosin (100 μm) and the quantification of the liver cell necrotic area. (d). Representative images of immunofluorescence analyses of F4/80 (200 μm) and the quantification of the positive area. (e). Hepatic mRNA expression of inflammation-related genes. *n* = 6 (sham), *n* = 5 (BDL), *n* = 6 (BDL+L. acidophilus) individuals/group. Data are expressed as the mean ± SEM. Two-tailed Student’s t-test or Mann–Whitney test. Columns with different letters differ significantly (*p* < 0.05). *p < 0.05, **p < 0.01. Scale bar: 100 μm or 200 μm. Abbreviations: *L. acidophilus*, *Lactobacillus acidophilus*.

To investigate the role of the *Lactobacillus* genus in cholestatic liver injury, we orally administered *L. acidophilus* after BDL in mice (Figure 4(a)). Compared with the sham group, serum ALT, AST, TBA, ALP, TBIL, and liver index were significantly increased in the BDL group, while *L. acidophilus* treatment significantly reduced (Figure 4(b), Supplementary Figure 4a-c), which is consistently with Mdr2^−/−^ mice (Supplementary Figure 5a-d). In addition, BDL mice showed severe hepatocyte necrosis, which was significantly improved by *L. acidophilus* treatment (Figure 4(c)). In liver tissue， BDL significantly elevated levels of hepatic F4/80 protein, mRNA levels of F4/80, TNF-α, IL-1β and IL-6, compared with Sham group, while were significantly attenuated by *L. acidophilus* treatment (Figure 4(d,e)). These results suggest that *L. acidophilus* treatment inhibited the liver inflammatory response of cholestatic and exhibited a protective effect.

### L. acidophilus inhibited BAs synthesis and enhanced BAs excretion

**Figure 5.**
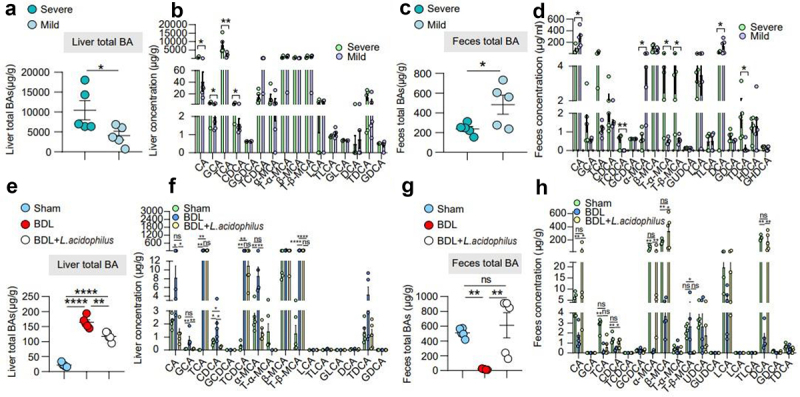
***L.*** *acidophilus* inhibited BAs synthesis and enhanced BAs excretion. (a)-(b). Liver BAs classes and BAs profile of mice with severe and mild injury. *n* = 6 individuals/group. (c)-(d). Feces BAs classes and BAs profile of mice with severe and mild injury. *n* = 6 individuals/group. (e)-(f). Liver BAs classes and BAs profile of *L. acidophilus* treatment. *n* = 6/5/6, respectively. (g)-(h). Feces BAs classes and BAs profile of *L. acidophilus* treatment. *n* = 6/5/6, respectively. Data are expressed as the mean ± SEM. Two-tailed Student’s t-test or Mann–Whitney test. Columns with different letters differ significantly (*P* < 0.05). * *P* < 0.05, ** *P* < 0.01, *** *P* < 0.001, **** *P* < 0.0001. Abbreviations: BAs, bile acids.

To identify the mechanism of *L. acidophilus* in cholestatic liver injury, we performed Kyoto encyclopedia of genes and genomes (KEGG) analysis and found that the pathways regarding BAs metabolism were involved in cholestatic liver injury (Supplementary Figure 6a). Therefore, we further finished liver tissue and fecal BAs profiles in the severe, mild, sham, BDL, and BDL+ *L. acidophilus* groups. The results showed that the levels of hepatic BAs were lower in the mild group than severe group (*p* < 0.05) (Figure 5(a)), with a predominant decrease in cholic acid (CA) (*p* < 0.05), and chenodeoxycholic acid (CDCA) (*p* < 0.05) (Figure 5(b)), while a higher fecal BAs excretion (*p* < 0.05) (Figure 5(c)), with a dominant increase in CA (*p* < 0.05), and deoxycholic acid (DCA) (*p* < 0.05) (Figure 5(d)). Similarly, we observed this phenomenon after *L. acidophilus* treatment in the BDL group (Figure 5(e-h)).

**Figure 6.**
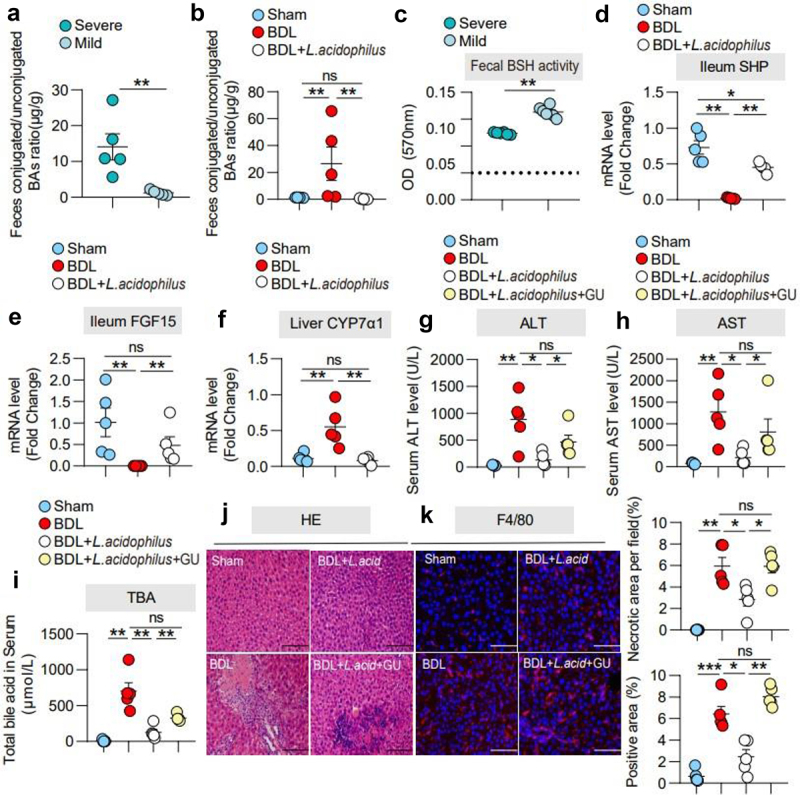
***L.*** *acidophilus* treatment ameliorated cholestatic liver injury is required FXR signaling. (a). The ratio of conjugated/unconjugated BAs in feces in severe and mild groups. *n* = 5 individuals/group. (b). The ratio of conjugated/unconjugated BAs in feces in the sham, BDL, and BDL+ *L. acidophilus* groups. *n* = 5 individuals/group. (c). Fecal BSH enzyme activity in the severe and mild groups. *n* = 5 individuals/group. (d)- (e). Ileum mRNA expression of SHP and FGF15. (f). Liver mRNA expression of CYP7α1. *n* = 5 individuals/group. (g)-(i). Serum ALT, AST and TBA levels. (j). Representative images of liver stained with hematoxylin and eosin (100 μm) and the quantification of the liver cell necrotic area. (k). Representative images of immunofluorescence analyses of F4/80 (200 μm) and the quantification of the positive area. Data are expressed as the mean ± SEM. Two-tailed Student’s t-test or Mann–Whitney test. Columns with different letters differ significantly (*P* < 0.05). * *P* < 0.05, ** *P* < 0.01. *** *P* < 0.001. Scale bar: 100 μm or 200 μm. Abbreviations: FXR, farnesoid X receptor; *L. acidophilus, Lactobacillus acidophilus;* GU, (z)-guggulsterone.

In the intestine, conjugated BAs may dissociate into unconjugated BAs through the action of intestinal bacteria for BAs excretion. The ratio of conjugated/unconjugated BAs is known to be regulated by BSH enzyme activity, and previous studies have shown that *Lactobacillus* has enriched BSH enzyme. Therefore, we examined BSH enzyme activity and the ratio of conjugated/unconjugated BAs in the severe and mild groups. The data showed that BSH enzyme activity was higher in the mild group (Figure 6(c)) and unconjugated BAs in feces were dominant compared with severe group (Figure 6(a)). Moreover, we found a similar phenomenon in the BDL + *L. acidophilus* group (Figure 6(b)).

Previous studies reported that the intestinal FXR- fibroblast growth factor 15 (FGF15) -hepatic CYP7α1 (the rate-limiting enzyme for BAs synthesis) axis is an important regulatory axis of BAs synthesis. BAs are known to activate FXR to induce FGF-15 expression in the intestine.^22–24^ In ileum tissue, we found that *L. acidophilus* partially recovered the expression of the FXR target gene small heterodimer partner (SHP) compared to the BDL group (Figure 6(d), Supplementary Figure 7a, b). In addition, BDL significantly reduced FGF-15 expression and elevated hepatic CYP7α1 expression, while *L. acidophilus* restored these effects (Figure 6(e,f)). To further clarify the role of FXR in the therapeutic effect of *L. acidophilus*, an inhibitor of FXR, GU ((Z)-guggulsterone), was used with or without *L. acidophilus*. Application of GU partially eliminated the protective effects of *L. acidophilus* on AST, ALT, TBA and hepatocyte necrosis, as well as the liver/body weight (%) after BDL (Figure 6(g-j)). In addition, inhibition of FXR by GU also attenuated the protective effect of *L. acidophilus* on the liver inflammatory response, including hepatic F4/80 protein, TNF-α, IL-6, IL-1β and F4/80 mRNA levels (Figure 6(k), Supplementary Figure 8a). Furthermore, GU significantly eliminated the effects of *L. acidophilus* on ileum SHP, FGF15 and hepatic CYP7α1 after BDL (Supplementary Figure 8b). These results further confirm that *L. acidophilus* inhibits hepatic BAs synthesis by activating intestinal FXR to inhibit liver CYP7α1.

### L. acidophilus promoted recovery in cholestatic liver injury patients

**Figure 7.**
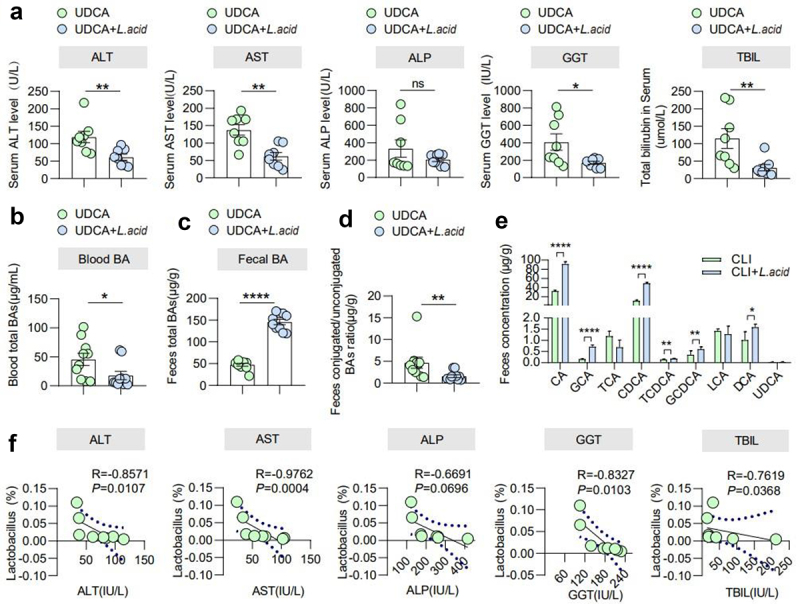
*L. acidophilus* promoted recovery in cholestatic liver injury patients. (a). Blood liver features include ALT, AST, ALP, GGT, and TBIL levels in the UDCA and UDCA+*L. acid* groups. *n* = 8 individuals/group. (b). Blood total BAs in the UDCA and UDCA+*L. acid* groups. *n* = 10 individuals/group. (c). Fecal BAs in the UDCA and UDCA+*L. acid* groups. *n* = 10 individuals/group. (d). The ratio of conjugated/unconjugated BAs in feces in the UDCA and UDCA+*L. acid* groups. *n* = 10 individuals/group. (e). Fecal BAs profile in the UDCA and UDCA+*L. acid* groups. *n* = 10 individuals/group. (f). The correlations between the abundance of *Lactobacillus* and ALT, AST, ALP, GGT, and TBIL levels were analyzed using Spearman’s correlation. *n* = 8 individuals/group. Data are expressed as the mean ± SEM. Two-tailed Student’s t-test or Mann–Whitney test. Columns with different letters differ significantly (*P* < 0.05). * *P* < 0.05, ** *P* < 0.01, *** *P* < 0.001, **** *P* < 0.0001.

To further explore and validate the effects of *L. acidophilus* in patients, A random-controlled clinical trial was performed with 20 enrolled patients suffering from cholestatic liver disease. The sample size was randomized and divided into control (n = 10) and treatment group (n = 10) (Supplementary Figure 9). In addition to the regular UDCA medication, the treatment group orally took a commercial tablet containing 5 × 10^6^ *L. acidophilus*, three times a day for 14 days. The baseline characteristics of all participants are shown in Supplemental Table 3. After 14 days, UDCA+*L. acid* group showed significantly lower AST (*p* < 0.05), ALT (*p* < 0.05), ALP, GGT (*p* < 0.05), and TBIL (*p* < 0.05) compared to UDCA (Figure 7(a)), while no differences were found at the beginning of the trial (Supplemental Table 3). Consistently, serum levels of FGF19 were higher in the UDCA+*L. acid* group (Supplementary Figure 10(a)). By analyzing the profile of BAs, the serum total BAs level significantly decreased and fecal BAs increased in UDCA+*L. acid* group compared to UDCA (*p* < 0.05) (Figure 7(b,c)). In addition, less conjugated BAs in feces were shown in UDCA+*L. acid* group (*p* < 0.05) (Figure 7(d)). Fecal 16S rRNA showed there were significant differences in β diversity after treatment (Supplementary Figure 11(a-d)), indicating that the gut microbiota composition was changed. Heatmap showed that the relative abundance of *Lactobacillus* was much higher in the UDCA+*L. acid* group (Supplementary Figure 12a-e). Correlation analysis showed that the relative abundance of *Lactobacillus* was negatively correlated with serum ALT (R = −0.8571, *p* = 0.0107), AST (R = −0.9762, *p* = 0.0004), ALP (R = −0.6691, *p* = 0.0696), GGT (R = −0.8327, *p* = 0.0103), and TBIL (R = −0.7619, *p* = 0.0368) (Figure 7(f)), implicating a beneficial relationship of *L. acidophilus* with liver function in cholestatic liver disease.

## Discussion

**Figure 8.**
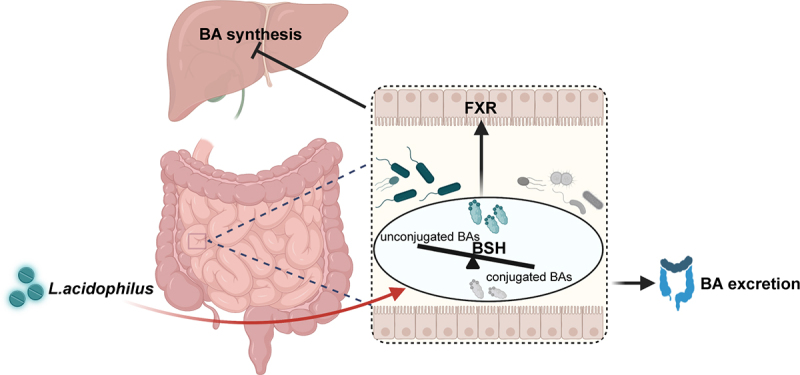
The schematic diagram of *L. acidophilus* ameliorating cholestatic liver injury. *L. acidophilus* inhibits hepatic BAs synthesis by activating intestinal FXR signaling, and increases unconjugated BAs by enriched BSH enzymes to enhance the BAs excretion, which could attenuate cholestatic liver injury.

Cholestatic liver disease, one of the major health problems affecting most people around the world, is not well treated despite increasing awareness. Strategies targeting gut microbiome-mediated treatment need to be explored.^25^ Herein, we showed that in mouse cholestatic liver injury induced by BDL, gut microbiota varied depending on the severity of liver injury, of which *L. acidophilus* was the most distinguishing genus. In our mouse model, we verified that *L. acidophilus* treatment inhibited BAs synthesis through decreased hepatic CYP7α1, restored ileum FGF15 and SHP on the FXR pathway; at the fecal side, *L. acidophilus* enriched in fecal BSH enzyme activity elevated unconjugated BAs, thus enhancing BAs excretion and resulting in the recovery of cholestatic liver injury. Furthermore, our small-sample randomized controlled clinical trial confirmed that supplementation of *L.* *acidophilus* could promote the recovery of liver function in cholestatic liver injury patients, associating with serum total BAs decrease and unconjugated BAs increase to enhance fecal BAs excretion (Figure 8).

Cholestatic liver disease is a complex clinical syndrome with intrahepatic or extrahepatic etiologies that results in BAs accumulation and aggravate liver injury, including PSC (Primary sclerosing cholangitis), PBC (Primary biliary cholangitis), and negative effects of drugs, such as non-steroidal anti-inflammatory drugs. If left untreated, chronic cholestasis may lead to liver fibrosis and cirrhosis.^26,27^ Recent studies suggest that gut microbiota plays a vital role in the pathophysiological process of cholestasis liver disease.^28–30^ Multidrug resistance protein (Mdr)^−/−^ knockout mice have shown dysbiosis of intestinal flora, which accelerated disease progression.^31^ Gut microbiota-macrophage interactions increase intestinal permeability and promote cholestatic liver disease.^32^ Our study confirmed that the severity of cholestatic liver injury is mediated in a gut microbiota-dependent manner, in which mice receiving feces from BDL-severe mice showed more significant liver tissue damage and a significant difference in microbial composition. The mild group showed enrichment of *Providencia*, *Acinetobacter*, *Enterobacter*, and *Lactobacillus*. Evidence has shown that *Lactobacillus* may be used in therapy of various diseases.^23^ For example, *Lactobacillus* significantly enhanced the efficacy of tumor resistance against cytotoxic T-lymphocyte-associated antigen 4 (CTLA-4)^33^ and prevented idiopathic eczema in infancy.^34^ In another study, administration of *L. acidophilus* provided pain relief^35^ and prevented antibiotic-associated diarrhea.^36^ Mice with cholestasis have shown obvious intestinal permeability and severity liver injury, while restored by *L. acidophilus* treatment. More importantly, our study also observed that *L. acidophilus* application alleviated cholestasis liver injury in cholestatic patients. Thus, there is a possibility that increased *L. acidophilus* could be responsible for mild cholestasis liver injury.

BAs homeostasis, including BAs synthesis and BAs excretion, is crucial for cholestasis progression. Regulating BAs metabolism is beneficial for cholestatic liver injury.^23^ BSH is a key factor in BAs metabolism,^37^ which can be regulated by gut microbe.^29^ Previous studies have demonstrated that *Lactobacillus* is enriched in BSH enzymatic activity, which may elevate unconjugated BAs level participating in BAs detoxification.^29,38,39^ Consistently, our findings showed that *L. acidophilus* increased fecal BAs excretion induced by higher BSH enzyme activity and more unconjugated BAs.

Farnesoid X receptor (FXR) is a ligand-activated transcription factor mainly expressed in the liver and intestine, which has been widely explored for its significant role in bile acid synthesis,^40^ BAs synthesis is regulated by enterohepatic signaling. Previous studies have demonstrated that FXR activation inhibits CYP7α1 and reduces BAs synthesis.^41,42^ Our findings demonstrated that inhibition of FXR could not improve cholestasis liver injury, suggesting that FXR is essential for the therapeutic effects of *L. acidophilus*. Consistent with a previous study,^3^ we also observed that the expression of FXR target gene in the ileum was activated and liver CYP7α1 was inhibited by treatment with *L. acidophilus*. Normally, BAs activate intestinal FXR and subsequently elevate the FGF15, which binds to the FGF receptor/β-Klotho complex on hepatocytes and inhibits BAs synthesis.^43^ Under BDL condition, ileum FGF15 is downregulated and liver CYP7α1 level is elevated but could be restored after *L. acidophilus* colonization. This finding has been confirmed with the Liver BAs profile. This phenomenon is most likely due to the circulation of BAs from high concentrations into the intestine.

Globally, several pre-clinical trials in *L. acidophilus* application have been conducted, including chronic cholestasis in children, diabetes, oral candidiasis associated with radiation therapy, and *Clostridium difficile* infections (https://www.clinicaltrials.gov/). It has been confirmed that *L. acidophilus* has a good acceptability and safety profile. Consistently, in our clinical trial, we observed a significant decrease in blood liver function indexes after *L. acidophilus* treatment, indicating prompt liver injury recovery progression. Similar to the results in mice, the BAs profiles after *L. acidophilus* treatment in patients showed relatively higher unconjugated BAs and enhanced BAs excretion. However, our clinical study has several limitations, including a small sample size and short-term treatment. Hence, clinical studies with larger sample sizes must be conducted before using *L. acidophilus* therapy in clinical practice.

Overall, our study demonstrates that *L. acidophilus* effectively inhibits hepatic BAs synthesis and enhances BAs excretion to attenuate cholestasis liver injury. These findings highlighted that gut microbiota-mediated BAs metabolism is a promising therapeutic modality and provides alternative management in cholestasis.

## Conclusions

In conclusion, our investigation attempts to provide information on gut microbiota in cholestatic liver injury. We found that *L. acidophilus* treatment could inhibit hepatic BAs synthesis and enhance fecal BAs excretion. The gut – liver axis plays an important role in cholestatic liver injury. Furthermore, the administration of *L. acidophilus* provides a novel therapeutic strategy to treat cholestatic liver injury.

## Supplementary Material

Supplemental Material

## Abbreviations

BDL: Bile duct ligation
ABX: Antibiotic cocktail
FMT: Fecal bacteria transplantation
Bas: Bile acids
*L. acidophilus*: *Lactobacillus acidophilus*
SCFAs: Short-chain fatty acids
PBC: Primary biliary cholangitis
PSC: Primary sclerosing cholangitis
LGG: *Lactobacillus rhamnosus GG*
CFUs: Colony-forming units
PBS: Phosphate buffered saline
OTUs: Operational taxonomy units
PCR: Polymerase chain reaction
ANOSIM: Analysis of similarities
PCoA: Principal coordinate analysis
LDA: linear discriminant analysis
LEfSe: linear discriminant analysis effect size
TNF- α: Tumor necrosis factor α
IL-6: Interleukin-6
IL-1β: Interleukin-1β
GAPDH: Glyceraldehyde 3-phosphate dehydrogenase
WT: Wild type
KEGG: Kyoto Encyclopedia of Genes and Genomes
FXR: Farnesoid X receptor
SHP: Small heterodimer partner
FGF15: Fibroblast growth factor 15
CYP7α1: Cholesterol 7α-hydroxylase
ALT: Serum alanine aminotransferase
AST: Aspartate aminotransferase
TBA: Total bile acid
ALP: Alkaline phosphatase
TBIL: Total bilirubin
CA: Cholic acid
CDCA: Chenodeoxycholic acid
DCA: Deoxycholic acid
UDCA: Ursodeoxycholic acid
CTLA-4: T-lymphocyte-associated antigen 4
GU: (Z)-guggulsterone
BSH: Bile salt hydrolase
Nrf2: Nuclear factor erythroid-derived 2-like-2
H&E: Hematoxylin and eosin

## Notes

The raw sequence data reported in this paper have been deposited in the Genome Sequence Archive (Genomics, Proteomics & Bioinformatics 2021) in National Genomics Data Center (Nucleic Acids Res 2022), China National Center for Bioinformation/Beijing Institute of Genomics, Chinese Academy of Sciences (GSA: CRA012996) that are publicly accessible at https://ngdc.cncb.ac.cn/gsa.
