# Supplementary material for: Lactobacillus acidophilus ameliorates cholestatic liver injury through inhibiting bile acid synthesis and promoting bile acid excretion
Source: Gut Microbes. 2024 Aug 29;16(1):2390176. doi: 10.1080/19490976.2024.2390176 (PMC11364073; doi:10.1080/19490976.2024.2390176)
Supplement: Supplemental Material [file KGMI_A_2390176_SM6923.zip › KGMI_A_2390176 (1)/supplementary material.docx]

***Lactobacillus acidophilus* ameliorates cholestatic liver injury through inhibiting bile acid synthesis and promoting bile acid excretion**

Lingyi Wu, Jianchun Zhou, An Zhou, Yuanyuan Lei, Li Tang, Shiping Hu, Sumin Wang, Xu Xiao, Qiao Chen, Dianji Tu, Cheng Lu, Yi Lai, Yiding Li, Xiao Zhang, Bo Tang, Shiming Yang


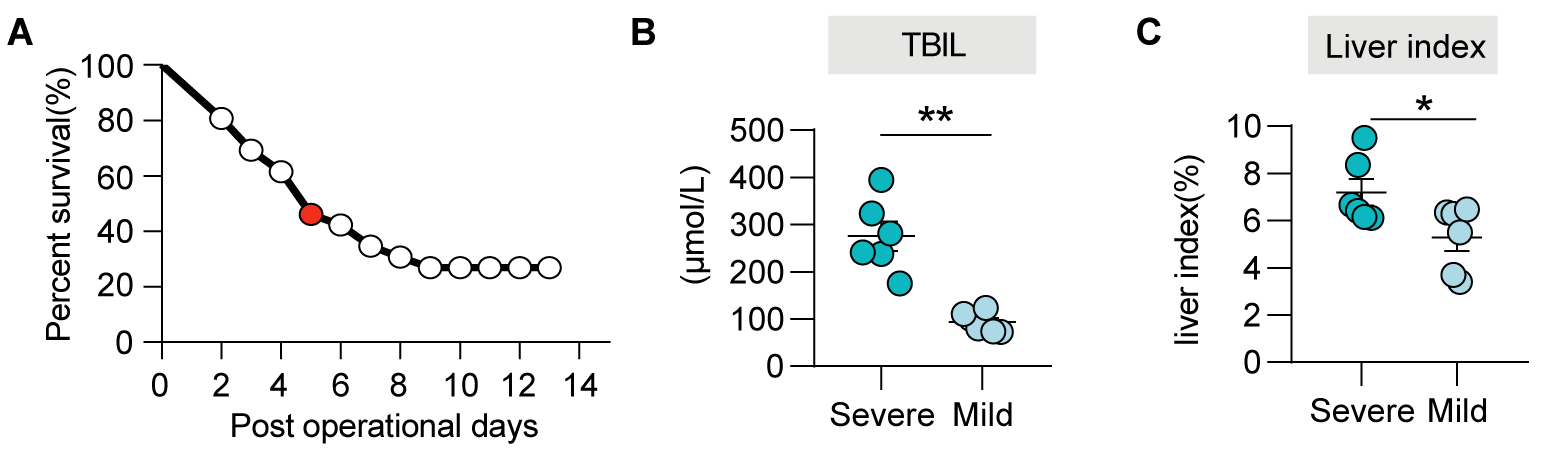


**Supplementary Fig. 1. Characterization of mice with BDL-induced injury.**

(A). The survival of mice after BDL was observed. All mice died at 30 days. The median survival time was 5 days. Mice were divided into two groups according to the cutoff line, and the groups showed different pathological characteristics. Therefore, mice that died at ≤ 5 days were classified as severe. (B). Serum TBIL levels. (C). Liver (g)/body weight (g) × 1%. *n* = 6 individuals/group. Data are expressed as the mean ± SEM. Columns with different letters differ significantly (*P* < 0.05). * *P* < 0.05, ** *P* < 0.01.


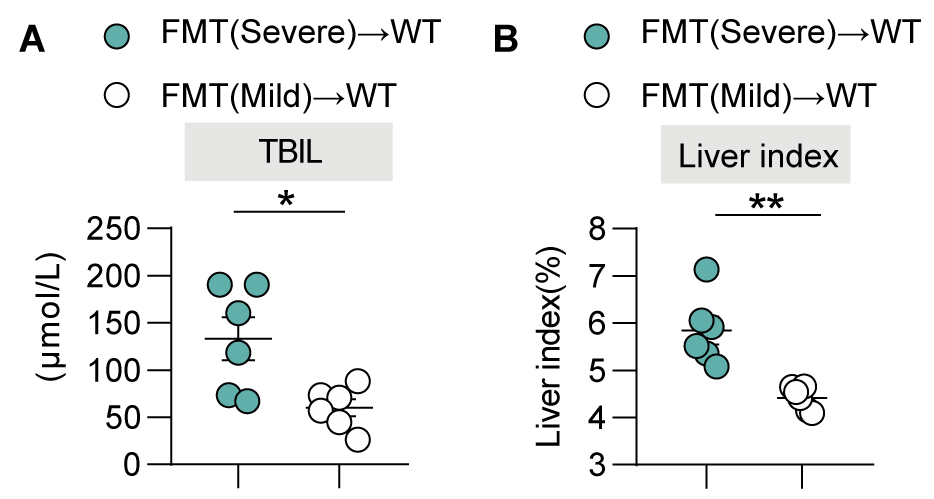


**Supplementary Fig. 2.** **The severity of cholestatic liver injury depends on the transmission of gut microbiota.** (A). Serum TBIL levels. (B). Liver (g)/body weight (g) × 1%. *n* = 6 individuals/group. Data are expressed as the mean ± SEM. Columns with different letters differ significantly (*P* < 0.05). * *P* < 0.05, ** *P* < 0.01.


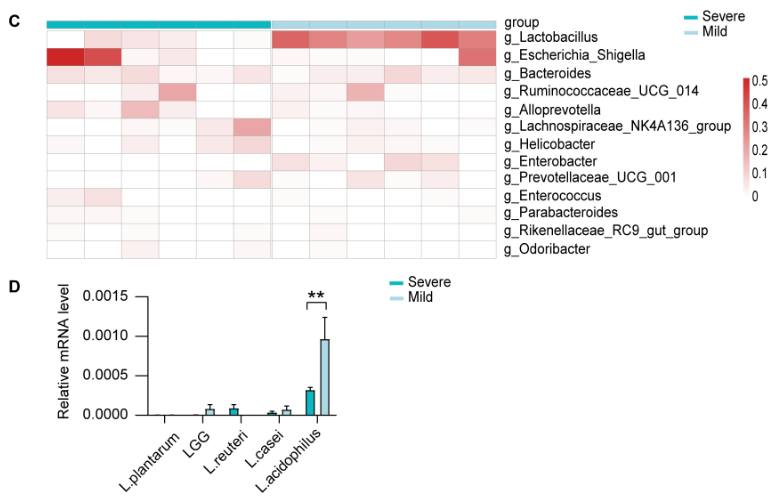

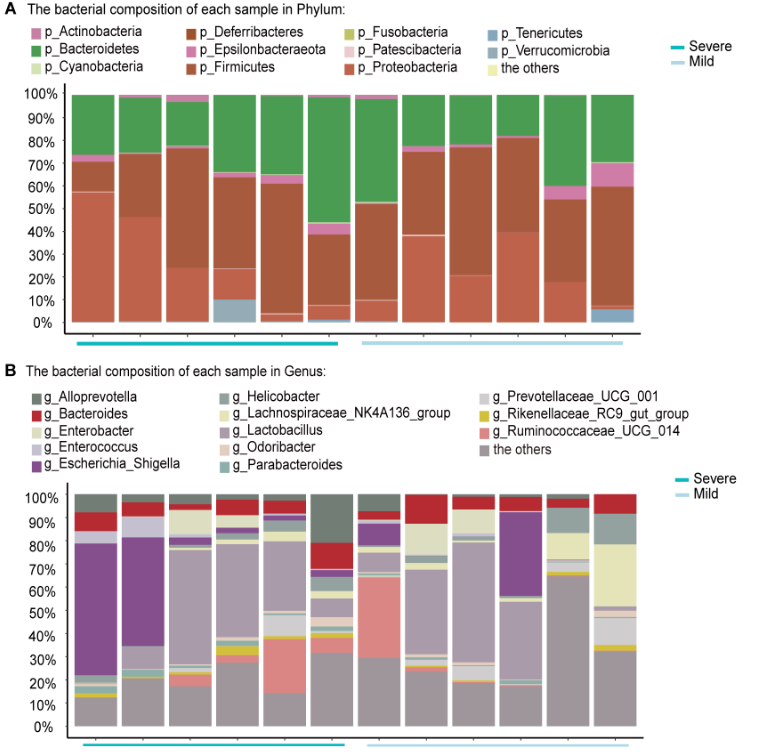


**Supplementary Fig. 3. Different BDL groups showed differences in gut microbiota.** (A). Bar plots of the phylum taxonomic levels in the two groups. (B). Bar plots of the genus taxonomic levels in the two groups. (C). Heatmap of selected differentially abundant features between mice with severe and mild injury. (D). Fecal mRNA levels of common bacterial subspecies of the genus *Lactobacillus. n* = 6 individuals/group.


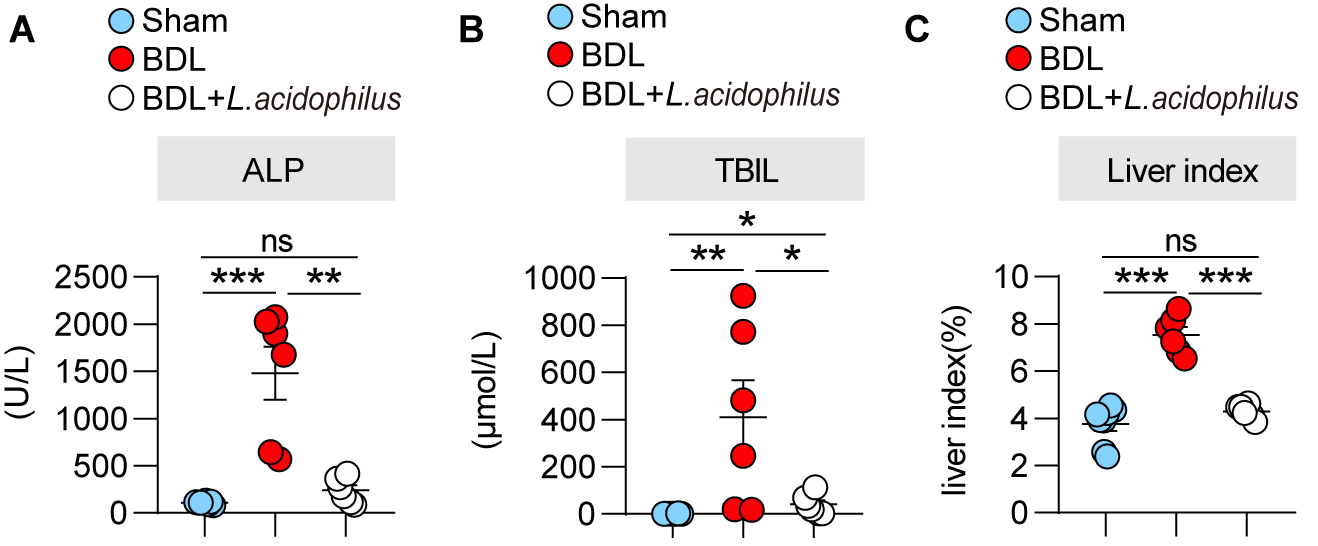


**Supplementary Fig. 4. Administration of *L. acidophilus* ameliorated cholestasis liver injury in cholestasis mice.** (A). Serum ALP levels. (B). Serum TBIL levels. (C). Liver (g)/body weight (g) × 1%. *n*= 6 individuals/group. Data are expressed as the mean ± SEM. Columns with different letters differ significantly (*P* < 0.05). * *P* < 0.05, ** *P* < 0.01.


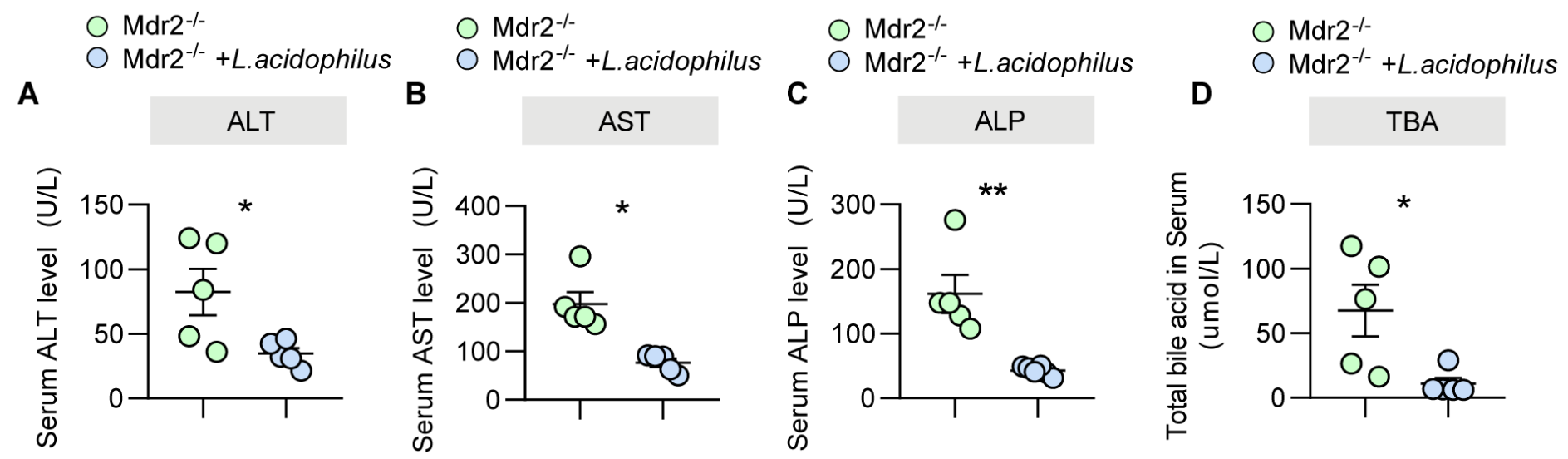


**Supplementary Fig. 5. Administration of *L. acidophilus* ameliorated cholestasis liver injury in Mdr2^-/-^ mice.** (A). Serum ALT levels. (B). Serum AST levels. (C). Serum ALP levels. (D). Serum TBA levels. *n*= 5 individuals/group. Data are expressed as the mean ± SEM. Columns with different letters differ significantly (*P* < 0.05). * *P* < 0.05, ** *P* < 0.01.


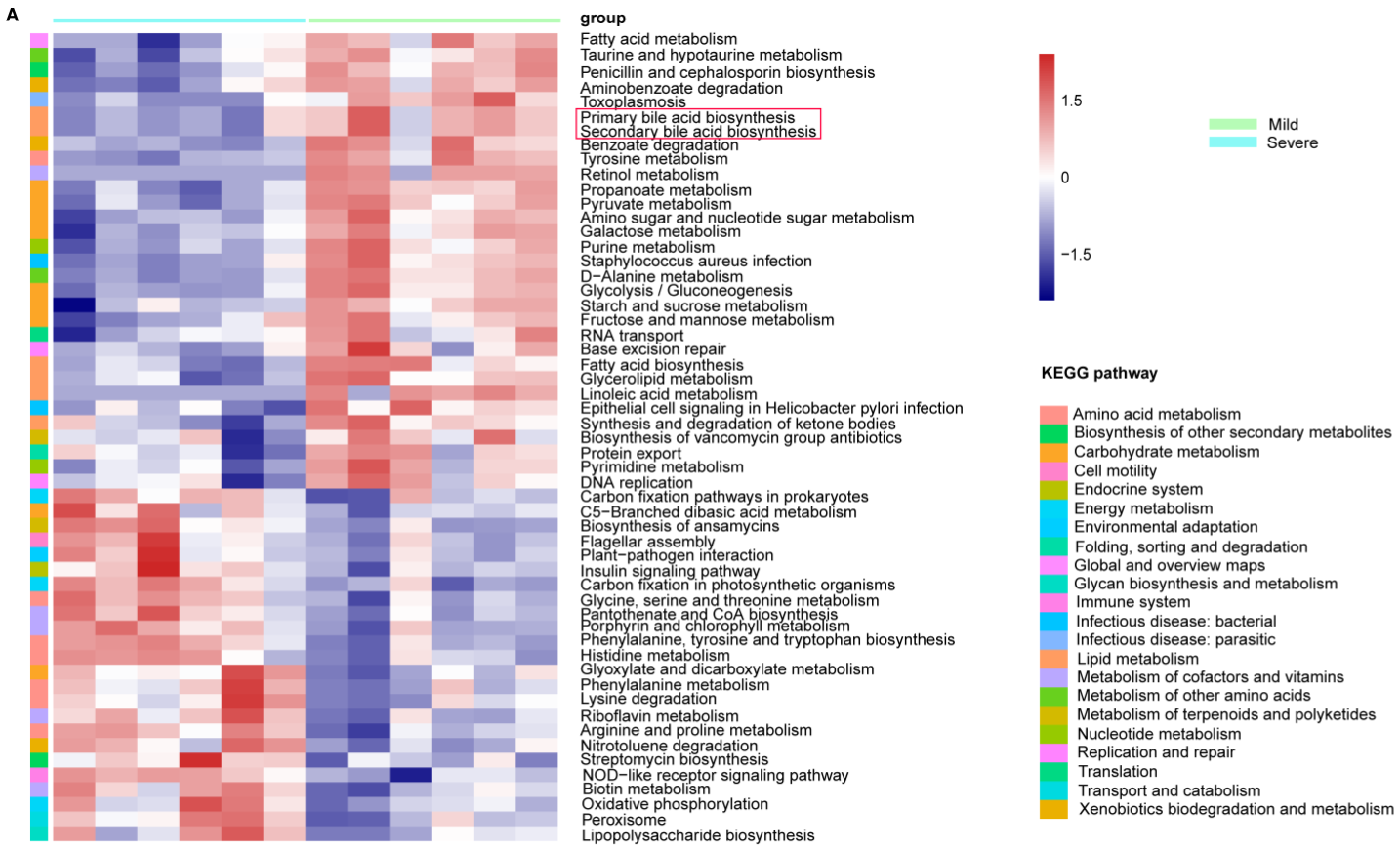


**Supplementary Fig. 6. KEGG analysis of mice with BDL-induced severe and mild injury.** (A). Annotation of microbial gene function of mice with severe and mild injury in KEGG pathway analysis. Analysis of 16S rRNA sequencing data from Fig. [3](https://microbiomejournal.biomedcentral.com/articles/10.1186/s40168-021-01065-2#Fig2). n = 6 individuals/group. Abbreviations: KEGG: Kyoto Encyclopedia of Genes and Genomes.


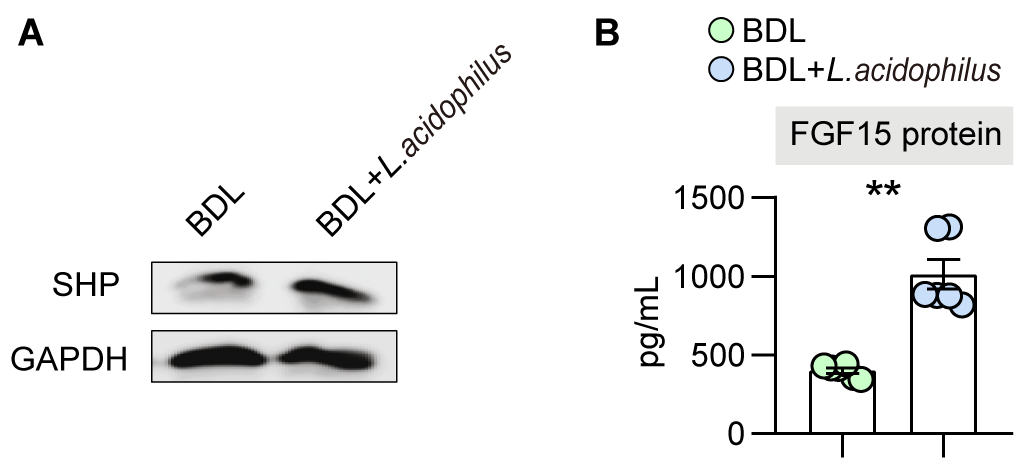


**Supplementary Fig. 7. Inhibition of global FXR activation attenuated the protective effects of *L. acidophilus* at the protein level.** (A). Western blot of SHP protein levels in ileum tissue of BDL and BDL+ *L. acidophilus* mice. (B). Elisa of FGF15 in ileum tissue of BDL and BDL+ *L. acidophilus* mice. *n*= 5 individuals/group. Data are expressed as the mean ± SEM. Columns with different letters differ significantly (*P* < 0.05). * *P* < 0.05, ** *P* < 0.01.


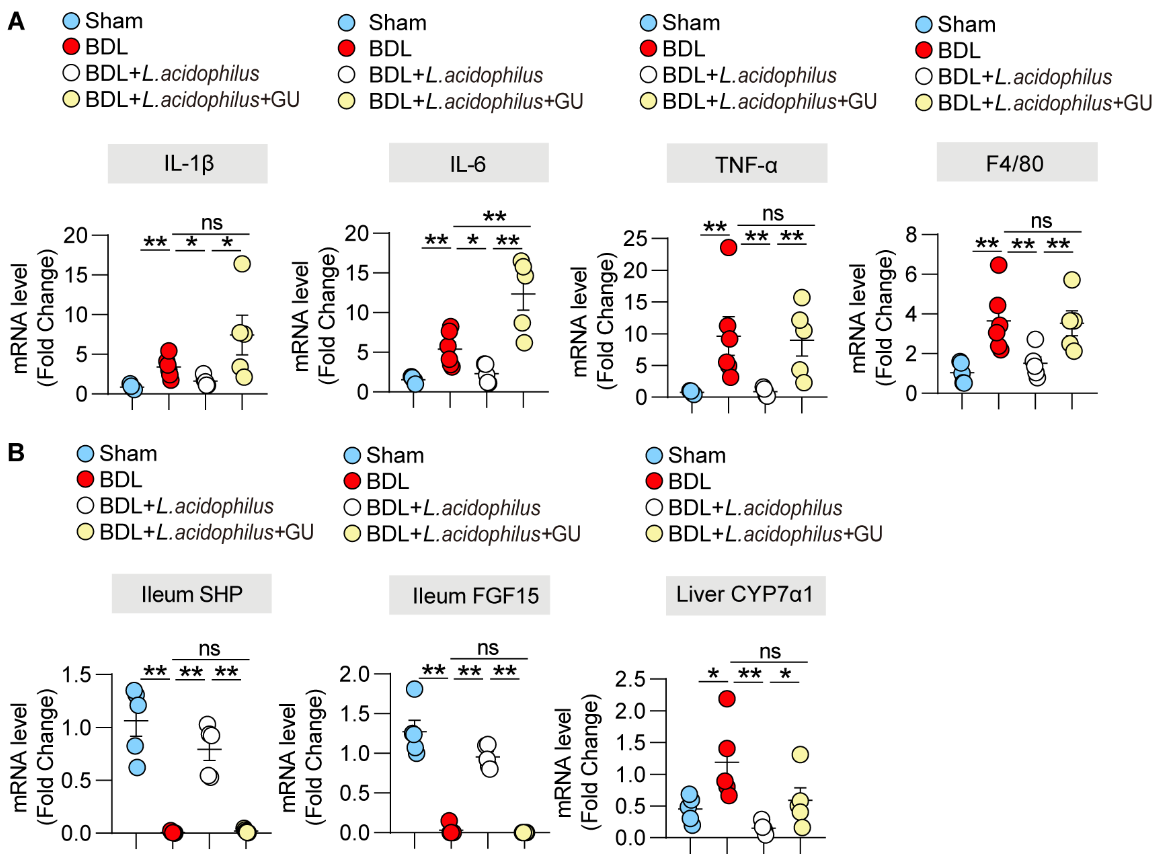


**Supplementary Fig. 8. *L. acidophilus* treatment ameliorated cholestatic liver injury is required FXR signaling.** (A). Hepatic mRNA expression of inflammation-related genes. (B). mRNA expression of ileum FGF15, SHP, and liver CYP7α1. *n* =6 (Sham), *n* =5 (BDL), *n* =6 (BDL+*L. acidophilus*), *n* =5 (BDL+*L. acidophilus+*GU) individuals/group. Data are expressed as the mean ± SEM. Columns with different letters differ significantly (*P* < 0.05). * *P* < 0.05, ** *P* < 0.01.


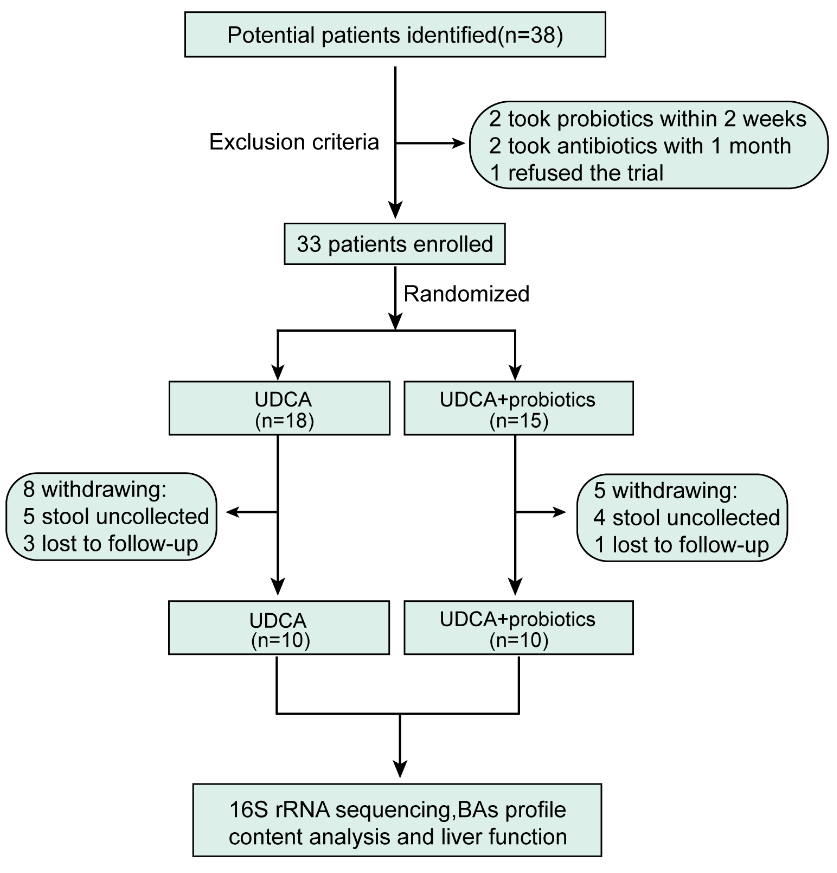


**Supplementary Fig. 9. Clinical trial enrollment flowchart.**


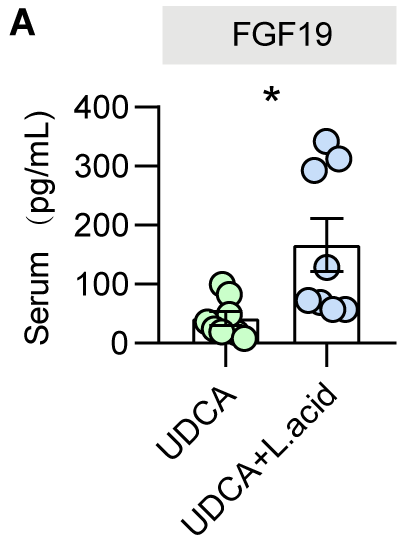


**Supplementary Fig. 10. FGF19 level.** (A). Serum levels of FGF19. n = 8 individuals/group.


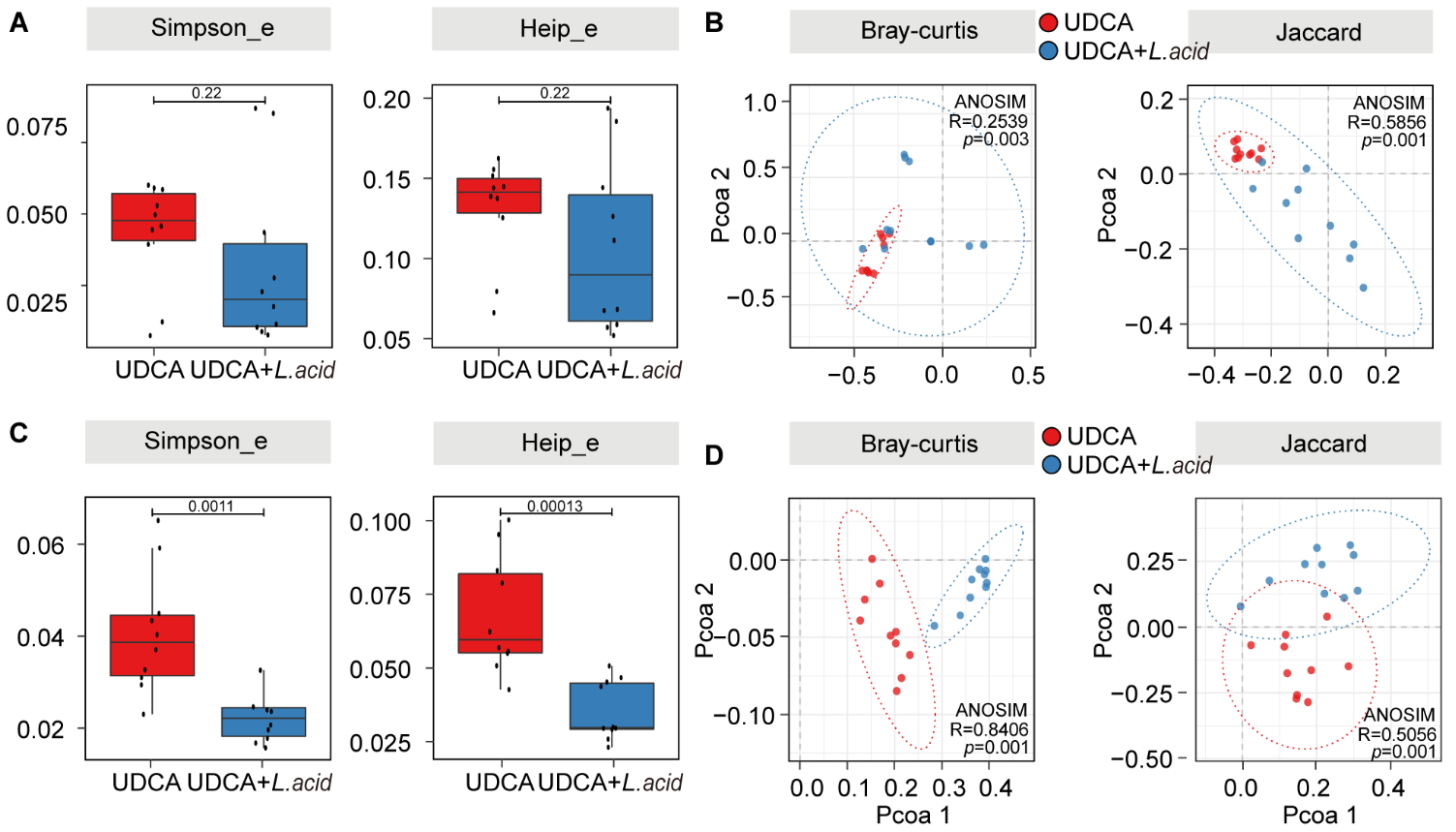


**Supplementary Fig. 11. Differences in gut microbiota between the UDCA and UDCA+*L. acid* groups.** (A). Alpha diversity in the two groups before the clinical trial, according to the Simpson_e and Heip_e diversity indices. (B). Bray-Curtis and Jaccard distance from the two groups at 0 days were determined by Unweighted UniFrac PCoA (principal coordinate analysis) of gut microbiota. (C). Alpha diversity in the two groups after 14 days, according to the Simpson_e and Heip_e diversity indices. (D). Bray-Curtis and Jaccard from the two groups at 14 days were determined by Unweighted UniFrac PCoA (principal coordinate analysis) of gut microbiota. n = 10 individuals/group.


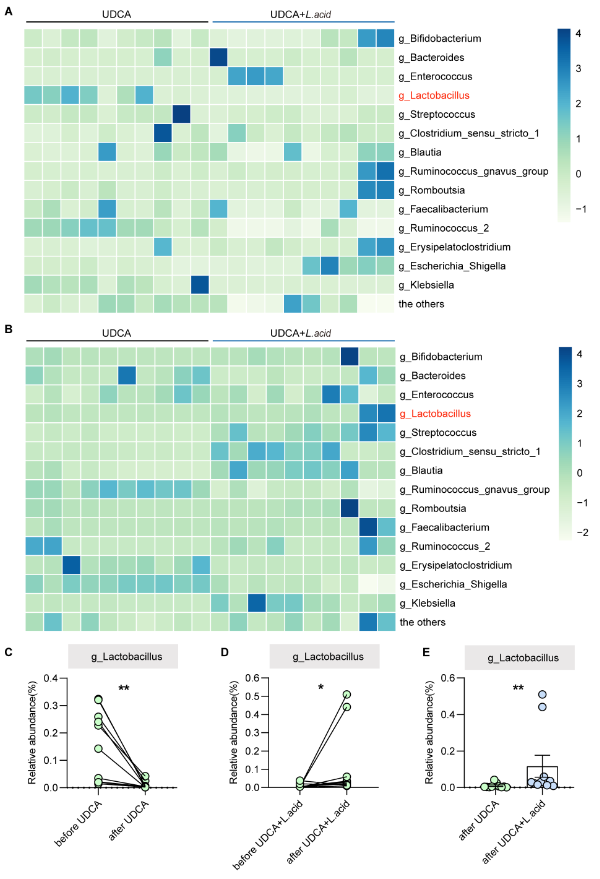


**Supplementary Fig. 12. Differences in gut microbiota between the UDCA and UDCA+*L. acid* groups.** (A). Heatmap of selected differentially abundant features between the UDCA and UDCA+*L. acid* groups at baseline. (B). Heatmap of selected differentially abundant features between the UDCA and UDCA+*L. acid* groups after treatment. (C)-(E). The relative abundance of *Lactobacillus. n* = 10 individuals/group.

**Supplementary Table 1. Primer sequences**

| Target genes | Sequences |
| --- | --- |
| 18S | Forward: ATTGGAGGGCAAGTCTGGTG |
|  | Reverse: CCGATCCCTAGTCGGCATAG |
| GAPDH | Forward: ACCCAGAAGACTGTGGATGG |
|  | Reverse: CACATTGGGGGTAGGAACAC |
| CYP7α1 | Forward: TGGGCATCTCAAGCAAACAC |
|  | Reverse: TCATTGCTTCAGGGCTCCTG |
| SHP | Forward: TCTGCAGGTCGTCCGACTATTC |
|  | Reverse: AGGCAGTGGCTGTGAGATGC |
| FXR | Forward: TGGGCTCCGAATCCTCTTAGA |
|  | Reverse: TGGTCCTCAAATAAGATCCTTGG |
| TNF-α | Forward: GCCTCCCTCTCATCAGTTCT |
|  | Reverse: CACTTGGTGGTTTGCTACGA |
| IL-1β | Forward: CTTTGAAGTTGACGGACCC |
|  | Reverse: TAGGTGATACTGCCTGCCTG |
| IL-6 | Forward: GAGGATACCACTCCCAACAGACC |
|  | Reverse: AAGTGCATCATCGTTGTTCATACA |
| FGF15 | Forward: ATGGCGAGAAAGTGGAACGG |
|  | Reverse: GGACCAGCGGAGTACAGGT |
| F4/80 | Forward: TGACTCACCTTGTGGTCCTAA |
|  | Reverse: CTTCCCAGAATCCAGTCTTTCC |

**Supplementary Table 2. Primers for *Lactobacillus* species.**

| Target genes | Sequences |
| --- | --- |
| ***Lactobacillus*** | Forward: GGACAGGTAGAAAGTCAAACGA |
| ***rhamnosus*** | Reverse: GCTGACCCGTAAACGCAATCTTAG |
| ***Lactobacillus*** | Forward: TTTGAGGGGACGACCCTCAAGCA |
| ***casei*** | Reverse: CGCCGACAAGCTATGAATTCACTTG |
| ***Lactobacillus*** | Forward: GGATTGGGTTTTGCGTGATGGTCGC |
| ***salivarius*** | Reverse: TGCATTTCCCCGCTTTCATGACT |
| ***Lactobacillus*** | Forward: CAGACAATCTTTGATTGTTTAG |
| ***brevis*** | Reverse: GCTTGTTGGTTTGGGCTCTTC |
| ***Lactobacillus*** | Forward: TGCAAAGTGGTAGCGTAAGC |
| ***acidophilus*** | Reverse: CCTTTCCCTCACGGTACTG  Probe: TACCACTTTGCAGTCCTACA |
| ***Lactobacillus*** | Forward: TGGATCACCTCCTTTCTAAGGAAT |
| ***plantarum*** | Reverse: TGTTCTCGGTTTCATTATGAAAAAATA |

**Supplementary Table 3. Baseline characteristics of patients in the two group**

| Characteristics | With  Probiotics (n=10) | Without  Probiotics (n=10) | *P* Value |
| --- | --- | --- | --- |
| Gender |  |  | *P*＞0.05 |
| Male | 4（40%） | 6（60%） |  |
| Female | 6（60%） | 4（40%） |  |
| Age (year) | 43.8±6.8 | 60.5±2.3 | *P*＞0.05 |
| m^2^ | 1.5±0.0 | 1.6±0.1 | *P*＞0.05 |
| Smoking | 2（20%） | 5（30%） | *P*＞0.05 |
| Alcohol | 1（10%） | 3（30%） | *P*＞0.05 |
| ALT (U/L) | 269.3±109.0 | 266.0±128.3 | *P*＞0.05 |
| AST (U/L) | 195.8±67.6 | 376.0±230.2 | *P*＞0.05 |
| ALP (U/L) | 348.2±56.76 | 365.7±82.56 | *P*＞0.05 |
| GGT (U/L) | 418.8±61.9 | 609.7±160.6 | *P*＞0.05 |
| TBIL (μmol/L) | 129.8±46.34 | 137.6±32.4 | *P*＞0.05 |

Data were represented as mean ± SEM.
