# Supplementary figures and images for: Lactobacillus acidophilus ameliorates cholestatic liver injury through inhibiting bile acid synthesis and promoting bile acid excretion
Source: Gut Microbes. 2024 Aug 29;16(1):2390176. doi: 10.1080/19490976.2024.2390176 (PMC11364073; doi:10.1080/19490976.2024.2390176)

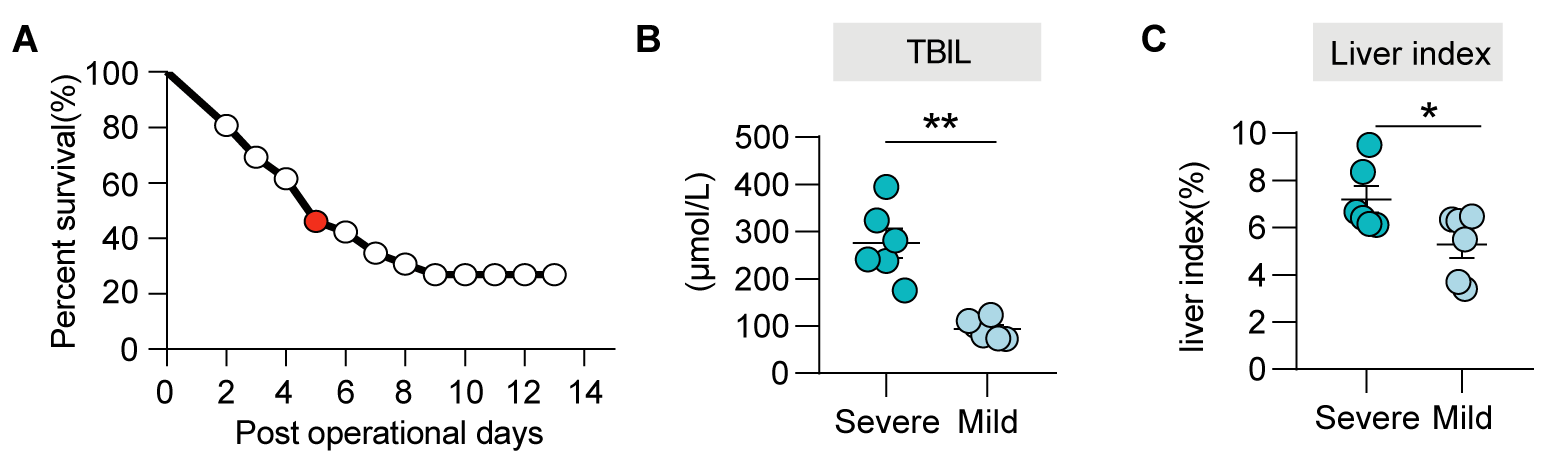

Supplement: Supplemental Material [file KGMI_A_2390176_SM6923.zip › KGMI_A_2390176 (1)/supplementary fig1.tif]

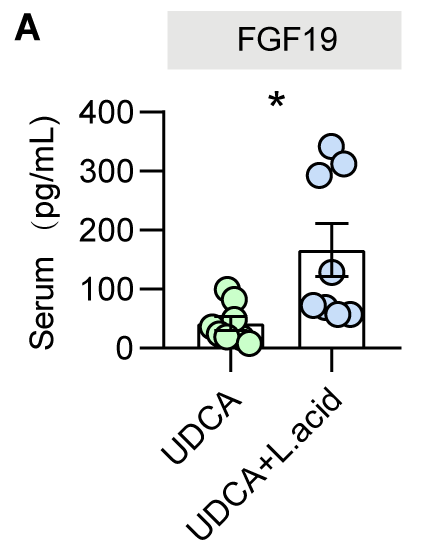

Supplement: Supplemental Material [file KGMI_A_2390176_SM6923.zip › KGMI_A_2390176 (1)/supplementary fig10.tif]

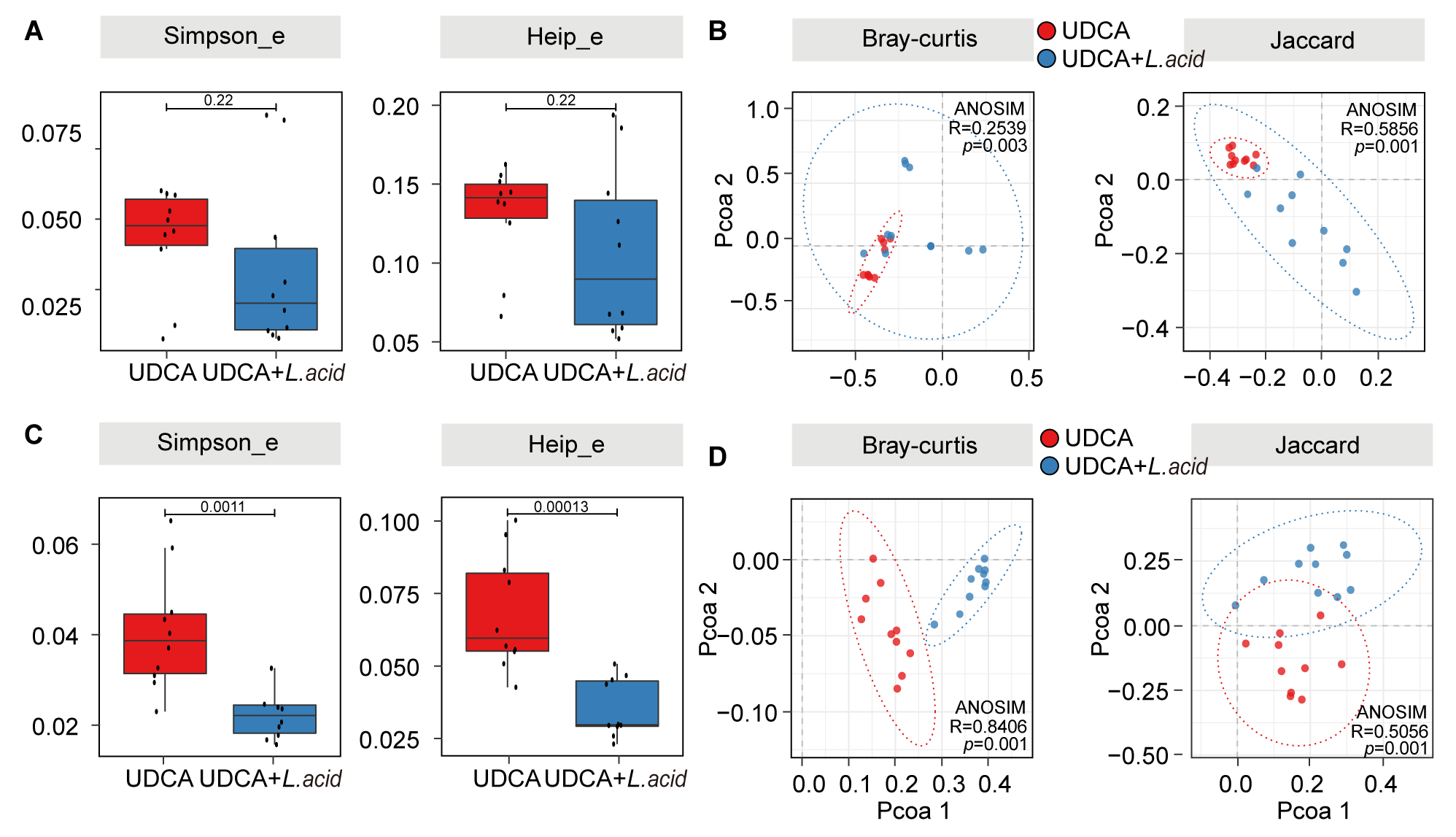

Supplement: Supplemental Material [file KGMI_A_2390176_SM6923.zip › KGMI_A_2390176 (1)/supplementary fig11.tif]

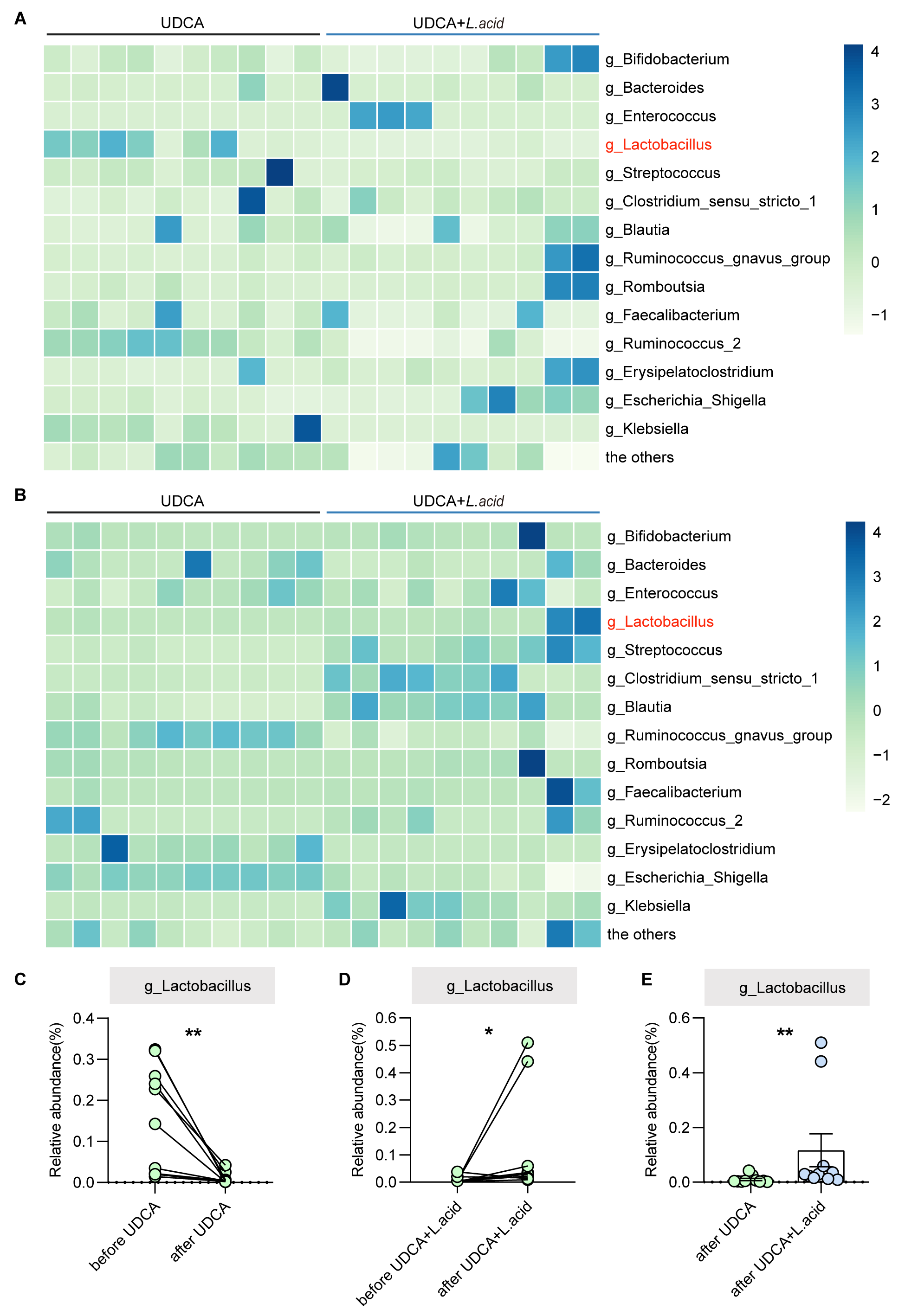

Supplement: Supplemental Material [file KGMI_A_2390176_SM6923.zip › KGMI_A_2390176 (1)/supplementary fig12.tif]

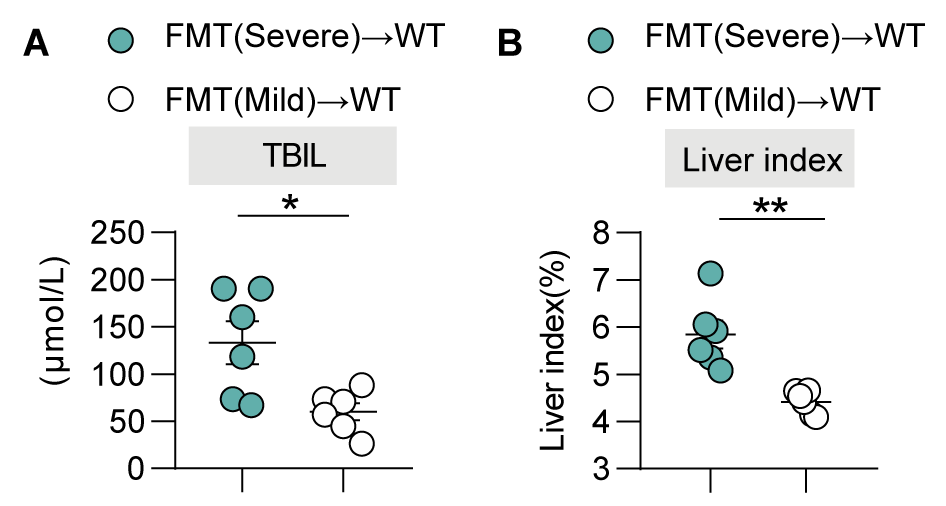

Supplement: Supplemental Material [file KGMI_A_2390176_SM6923.zip › KGMI_A_2390176 (1)/supplementary fig2.tif]

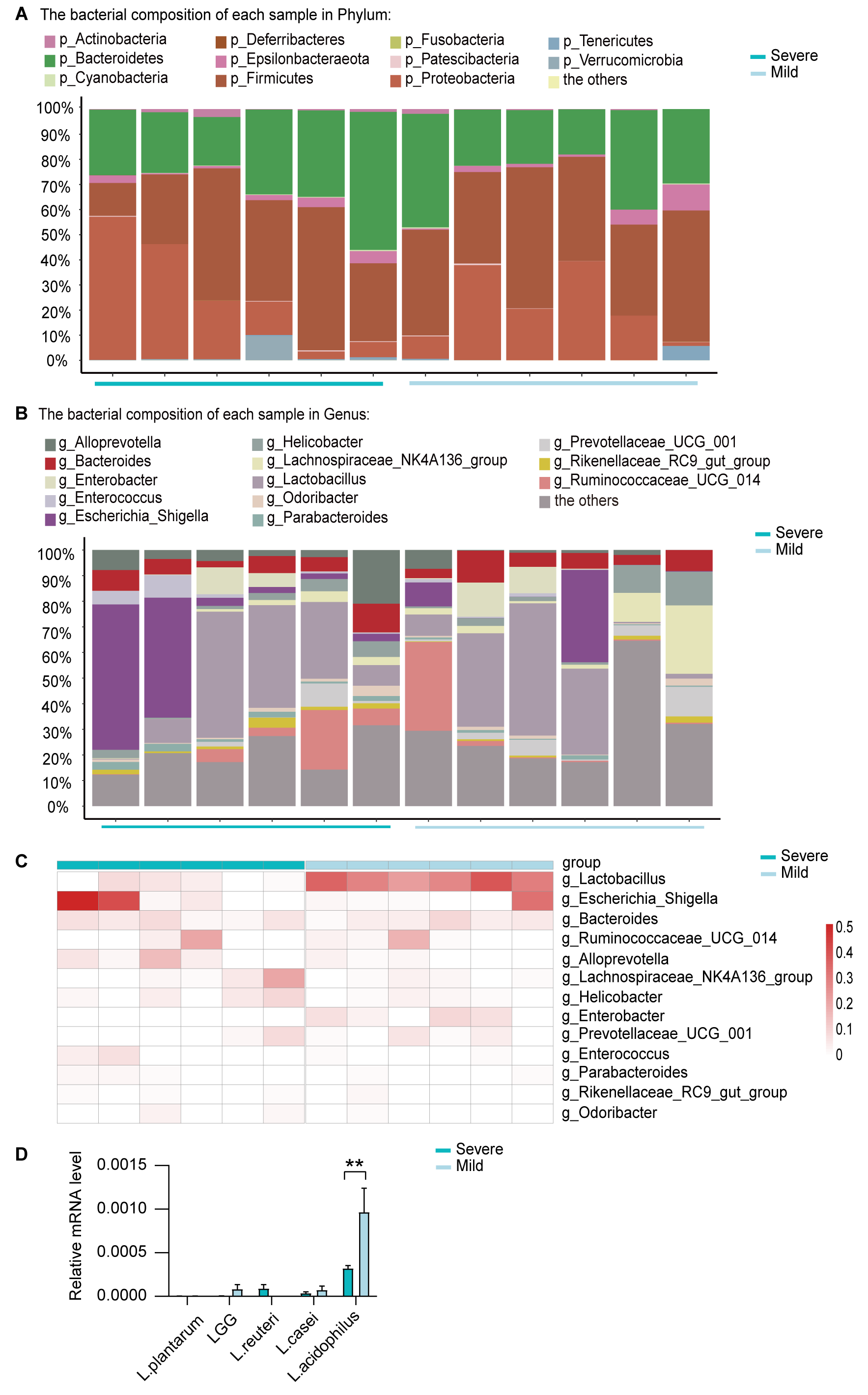

Supplement: Supplemental Material [file KGMI_A_2390176_SM6923.zip › KGMI_A_2390176 (1)/supplementary fig3.tif]

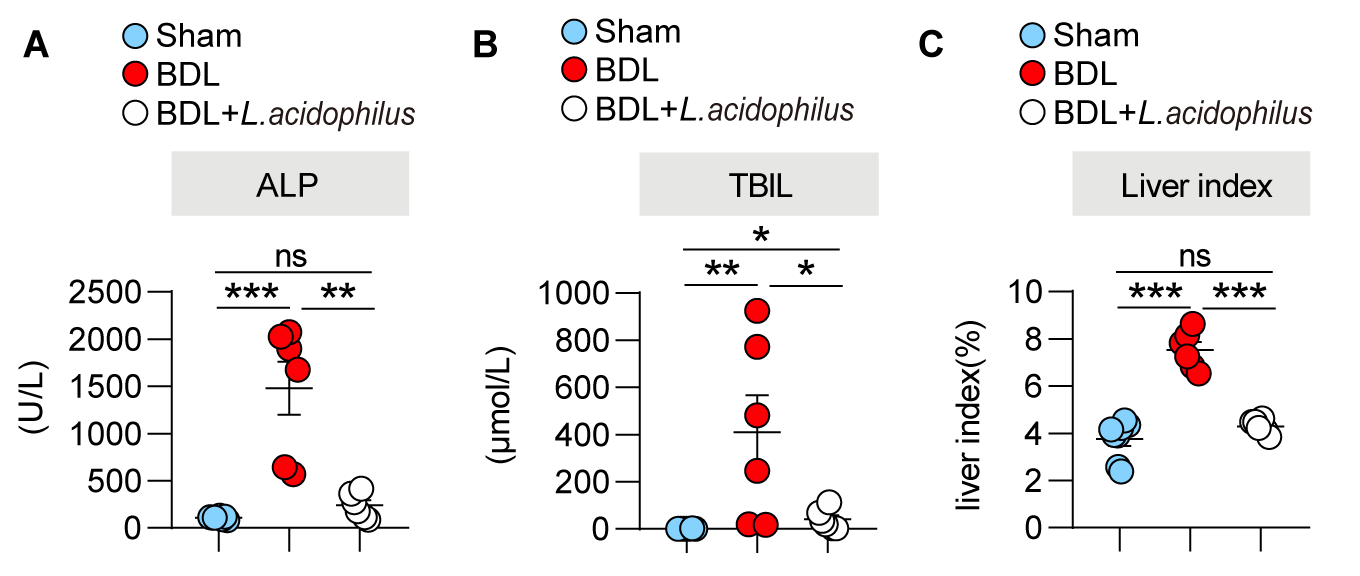

Supplement: Supplemental Material [file KGMI_A_2390176_SM6923.zip › KGMI_A_2390176 (1)/supplementary fig4.tif]

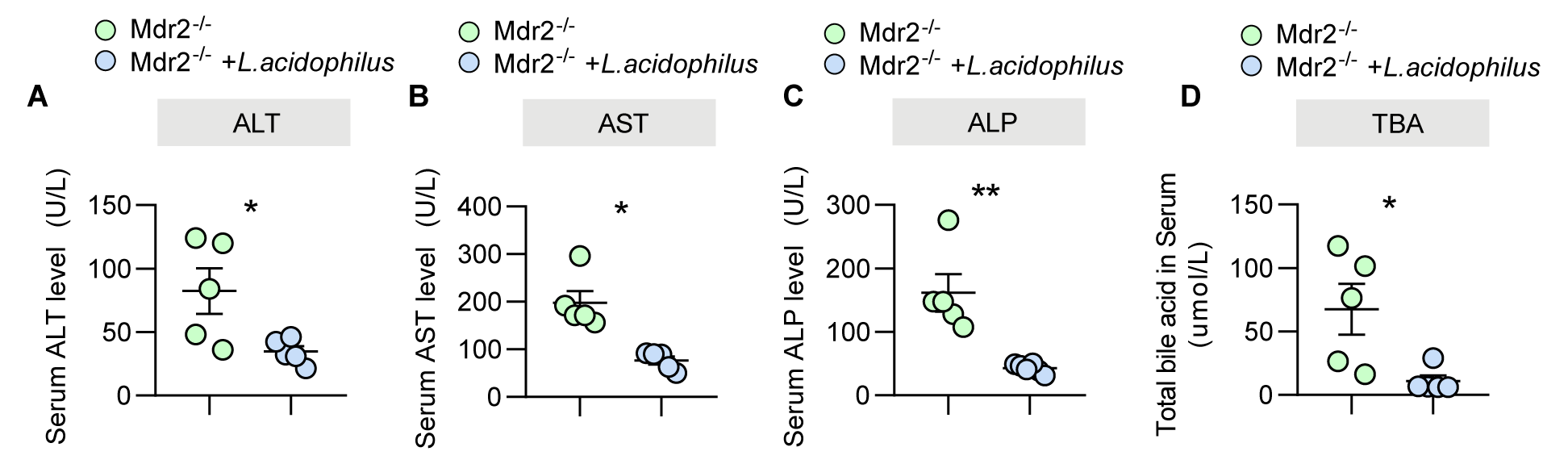

Supplement: Supplemental Material [file KGMI_A_2390176_SM6923.zip › KGMI_A_2390176 (1)/supplementary fig5.tif]

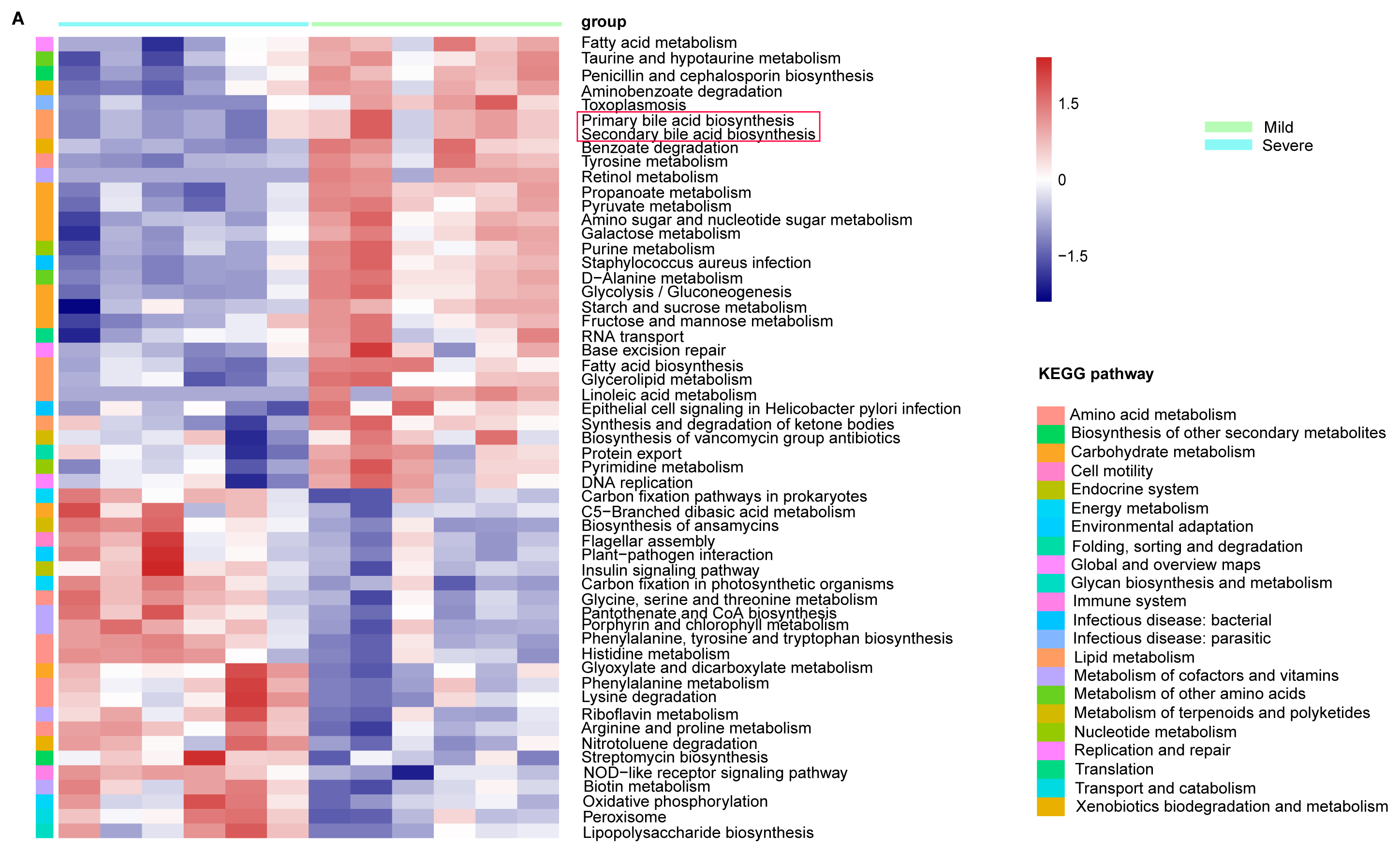

Supplement: Supplemental Material [file KGMI_A_2390176_SM6923.zip › KGMI_A_2390176 (1)/supplementary fig6.tif]

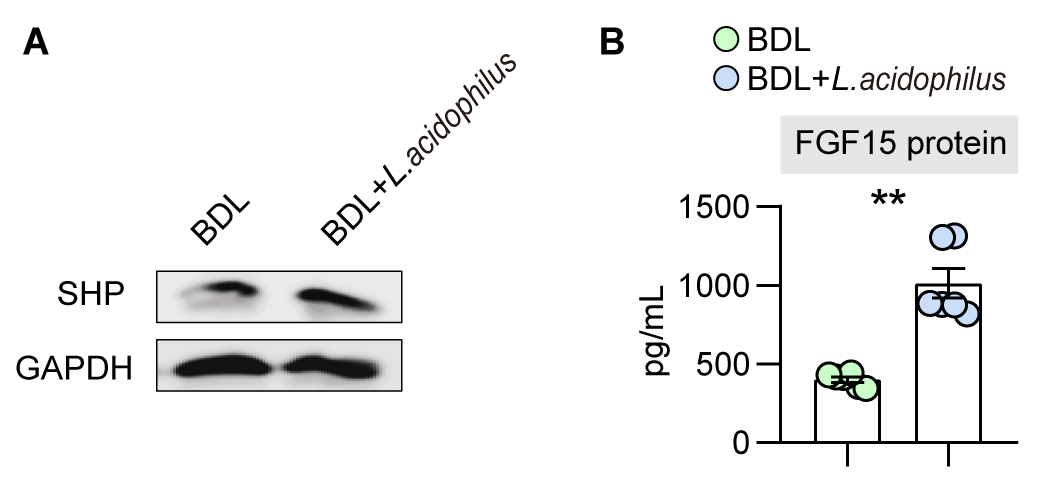

Supplement: Supplemental Material [file KGMI_A_2390176_SM6923.zip › KGMI_A_2390176 (1)/supplementary fig7.tif]

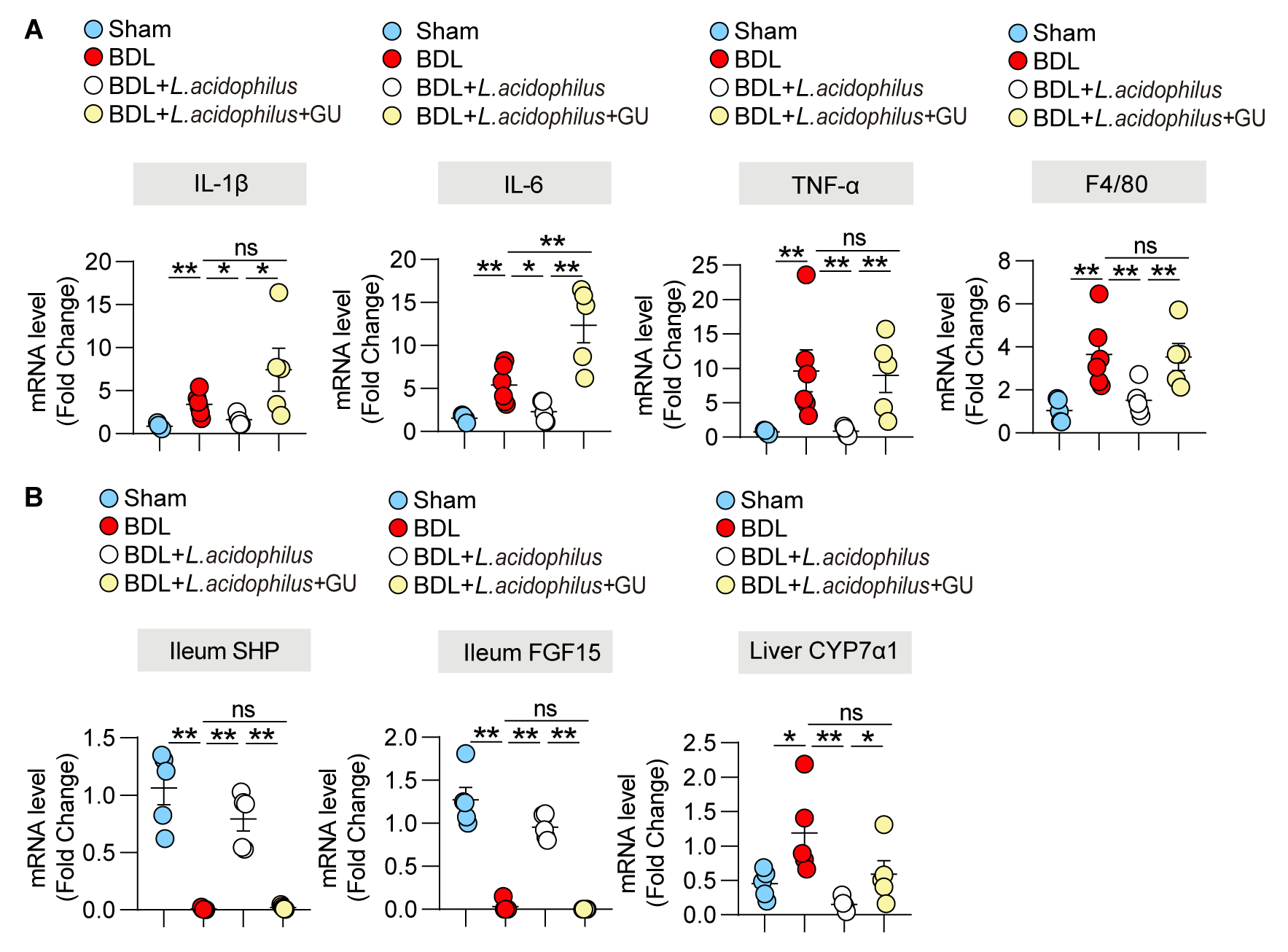

Supplement: Supplemental Material [file KGMI_A_2390176_SM6923.zip › KGMI_A_2390176 (1)/supplementary fig8.tif]

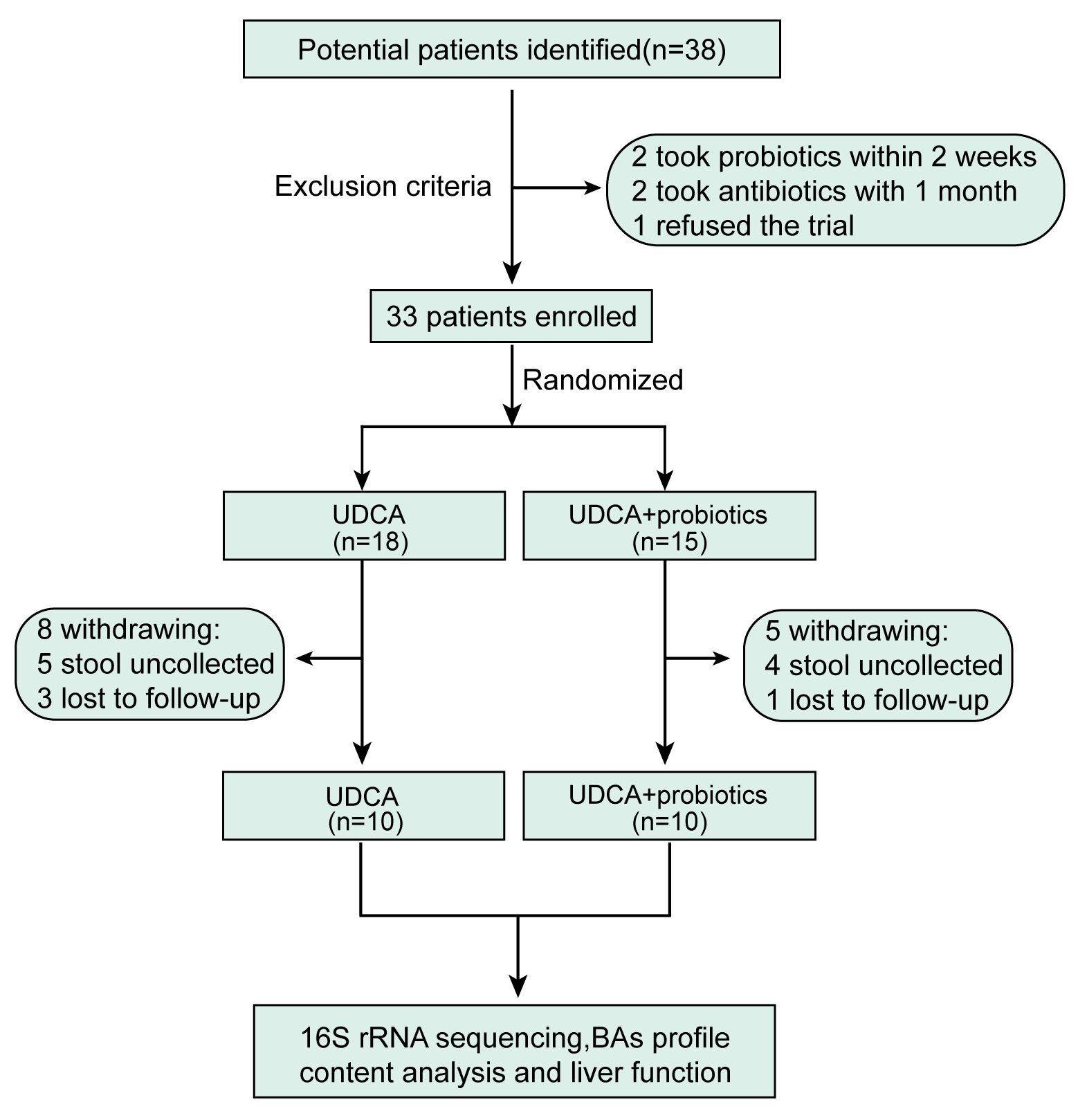

Supplement: Supplemental Material [file KGMI_A_2390176_SM6923.zip › KGMI_A_2390176 (1)/supplementary fig9.tif]

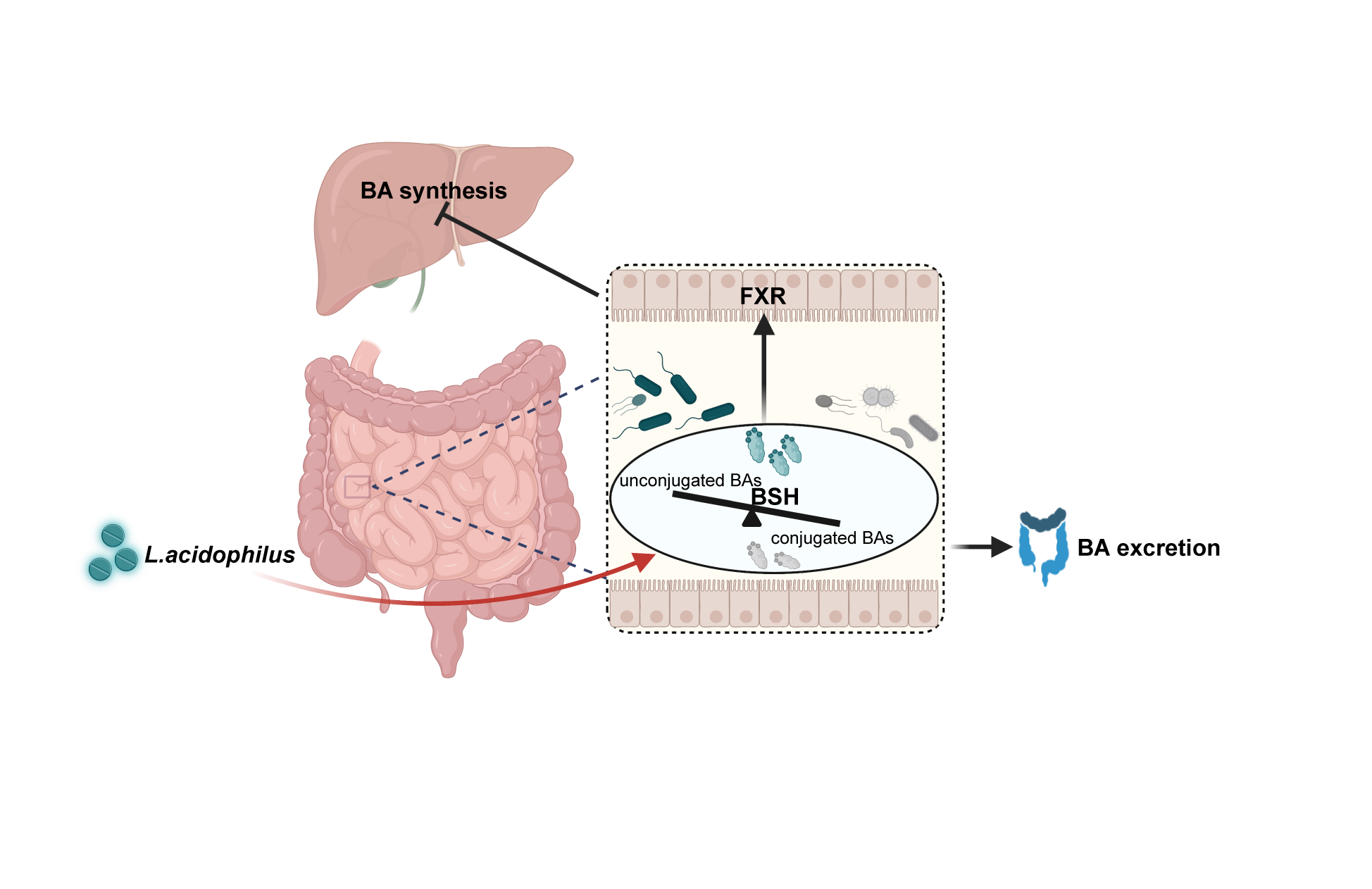

Supplement: Supplemental Material [file KGMI_A_2390176_SM6923.zip › KGMI_A_2390176 (1)/supplementary graphical abstract.tif]
